# BnUC1 Is a Key Regulator of Epidermal Wax Biosynthesis and Lipid Transport in *Brassica napus*

**DOI:** 10.3390/ijms25179533

**Published:** 2024-09-02

**Authors:** Fei Ni, Mao Yang, Jun Chen, Yifei Guo, Shubei Wan, Zisu Zhao, Sijie Yang, Lingna Kong, Pu Chu, Rongzhan Guan

**Affiliations:** State Key Laboratory of Crop Genetics & Germplasm Enhancement and Utilization, Jiangsu Collaborative Innovation Center for Modern Crop Production, College of Agriculture, Nanjing Agricultural University, Nanjing 210095, China; 2019201051@njau.edu.cn (F.N.); yangmao@njau.edu.cn (M.Y.); 2020101081@stu.njau.edu.cn (J.C.); 2022101138@stu.njau.edu.cn (Y.G.); 2016201079@njau.edu.cn (S.W.); 2020201042@stu.njau.edu.cn (Z.Z.); 2021101077@stu.njau.edu.cn (S.Y.); klnjj@njau.edu.cn (L.K.); chupu@njau.edu.cn (P.C.)

**Keywords:** *Brassica napus*, BnUC1, epidermal wax, bHLH

## Abstract

The bHLH (basic helix–loop–helix) transcription factor AtCFLAP2 regulates epidermal wax accumulation, but the underlying molecular mechanism remains unknown. We obtained *BnUC1^mut^* (*BnaA05g18250D* homologous to *AtCFLAP2*) from a *Brassica napus* mutant with up-curling leaves (*Bnuc1*) and epidermal wax deficiency via map-based cloning. BnUC1^mut^ contains a point mutation (N200S) in the conserved dimerization domain. Overexpressing *BnUC1^mut^* in ZS11 (Zhongshuang11) significantly decreased the leaf epidermal wax content, resulting in up-curled and glossy leaves. In contrast, knocking out *BnUC1^mut^* in ZS11-NIL (Zhongshuang11-near-isogenic line) restored the normal leaf phenotype (i.e., flat) and significantly increased the leaf epidermal wax content. The point mutation weakens the ability of BnUC1^mut^ to bind to the promoters of VLCFA (very-long-chain fatty acids) synthesis-related genes, including *KCS* (*β*-ketoacyl coenzyme synthase) and *LACS* (long-chain acyl CoA synthetase), as well as lipid transport-related genes, including *LTP* (non-specific lipid transfer protein). The resulting sharp decrease in the transcription of genes affecting VLCFA biosynthesis and lipid transport disrupts the normal accumulation of leaf epidermal wax. Thus, BnUC1 influences epidermal wax formation by regulating the expression of *LTP* and genes associated with VLCFA biosynthesis. Our findings provide a foundation for future investigations on the mechanism mediating plant epidermal wax accumulation.

## 1. Introduction

The cuticle is composed of wax and cutin, which is a hydrophobic layer that covers the leaf epidermis, thereby protecting plants from biotic and abiotic stresses [1,2,3]. Cutin is primarily a polymer cross-linked with glycerol and long-chain fatty acids (C16 and C18) that provides mechanical strength to the outer layer. In contrast, epidermal wax is an organic mixture composed mainly of very-long-chain fatty acids (VLCFAs; C20 to C30) and their derivatives, including alkanes, aldehydes, primary and secondary alcohols, ketones, esters, and other compounds [1]. Because of epidermal wax, the epidermis of most plants has a white frost-like appearance; however, the loss of wax can lead to a glossy appearance, which is a desirable trait in some economically important crops [4].

The plant cuticular wax biosynthetic pathway has been studied extensively. The biosynthesis of cuticular wax begins with the de novo synthesis of C16 and C18 fatty acids in epidermal cell plastids. The elongation of fatty acids by the fatty acid elongase complex in the endoplasmic reticulum leads to the production of VLCFAs, which are subsequently converted to wax compounds [5]. Genes encoding 3-ketoacyl-CoA synthase (*KCS1*, *KCS2*, *KCS6*, and *KCS9*) and fatty acyl-CoA reductase (*FAR3*) are involved in the synthesis of fatty acid precursors [6,7,8,9]. The suppression of *KCS6* expression significantly decreases the formation of wax compounds with carbon chain lengths greater than 24, reflecting the importance of *KCS6* for the elongation of VLCFAs [10]. In *Arabidopsis thaliana*, genes encoding ECERIFERUM (*CER1*, *CER3*, and *CER16*) and midchain alkane hydroxylase (*MAH1*) are involved in the alkane biosynthetic pathway [11,12,13,14,15].

The transport of cuticular wax is primarily mediated by transporter proteins. A previous study on the *acbp1* (acyl-CoA-binding protein1) *A. thaliana* mutant detected a decrease in the total amount of wax on the stem, suggesting that ACBP1 localized to the endoplasmic reticulum, and cell membrane may contribute to the transport of wax components [16]. An earlier analysis of the *cer5* mutant indicated that the CER5 transporter protein facilitates the export of wax components [17]. In addition to the ATP-binding cassette transporters, lipid transfer proteins (LTPs) can bind and transport fatty acids and may be involved in transporting wax components out of the cell wall. According to recent research, *LTP* genes affect the formation of the cuticle, but they must be more thoroughly functionally characterized [18].

Wax biosynthesis is mainly regulated at three levels (transcriptional, post-transcriptional, and post-translational). Different types of transcription factors help regulate cuticle development, including AP2/ERF, MYB, and HD-ZIP transcription factors [19]. For example, in *A. thaliana*, WRI4, WIN1/SHINE1, and DEWAX2 are AP2 transcription factors related to wax biosynthesis [20,21]. Both MYB96 and HD-ZIP regulate epidermal wax metabolism [22,23]. As one of the important oil crops, *Brassica napus* has the characteristics of high oil content and excellent agronomic traits [24,25]. However, various biotic and abiotic stresses limit the further utilization of *B. napus*, and the cuticle of terrestrial plants forms a barrier against environmental stress [1,2,3,26]. A mutation in the *BnaA.GL* gene caused downregulation of cuticular wax biosynthetic genes and deficiency of cuticular wax in *B. napus* [27]. The mutations in the *FAR* (fatty acyl-CoA reductase) genes, specifically *BnA1.CER4* and *BnC1.CER4*, result in the formation of irregular wax crystal patterns and modifications to the composition of epidermal wax in *B. napus* [28]. Overexpression of *BnKCS1-1*, *BnKCS1-2*, and *BnCER1-2* promotes cuticular wax production and increases drought tolerance in *B. napus* [29]. Although research on epidermal wax is well known in a number of plant species, there is little research on the epidermal function in *B. napus* [5,18].

In this study, we identified *BnUC1* as a basic helix–loop–helix (bHLH) transcription factor gene associated with leaf cuticular wax accumulation and the up-curled leaf phenotype. An amino acid substitution in the conserved bHLH dimerization domain of BnUC1 was observed to alter the ability of the encoded transcription factor to control the expression of downstream genes associated with VLCFA biosynthesis as well as *LTP* genes. Because of this point mutation, BnUC1^mut^ was unable to bind to the promoters of wax synthesis-related genes and lipid transporter genes, leading to substantially decreased leaf epidermal wax production as well as leaf curling in *B. napus*.

## 2. Results

### 2.1. Cloning and Expression Pattern Analysis of BnUC1

**Figure 1:**
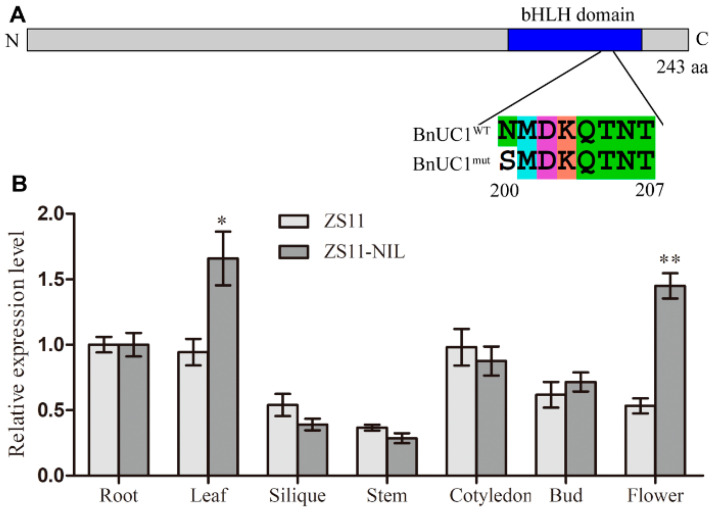
Cloning and expression pattern analysis of *BnUC1* from the mapping parents. (**A**) The point mutation (N200S) of BnUC1 in mapping parent ZS11 (BnUC1^WT^) and ZS11-NIL (BnUC1^mut^). The blue box represents conserved bHLH domain. (**B**) The gene expression pattern analysis for the *BnUC1* gene. Expression levels of *BnUC1* detected by qRT-PCR in various tissues, including roots, leaves, siliques, stems, cotyledons, buds, and flowers of ZS11 and ZS11-NIL. The *BnActin7* was used as the internal reference gene. * represents a significance level of less than 0.05, ** represents a significance level of less than 0.01.

We previously mapped a 54.8 kb interval on chromosome A05 in *B. napus*. This interval included *BnaA05g18250D* and *BnaA05g18290D* [4], which were predicted to be candidate genes responsible for the formation of leaf morphological characteristics. To further investigate the mechanism underlying the up-curled leaf trait, the two mapping parents were transformed with the two candidate genes (described in the following section). Based on previous localization studies, the locus of the up-curling leaf (named *Bnuc1*) is controlled by a dominant locus [4]. In this study, a candidate gene *BnaA05g18250D* was found to control leaf up-curling and named *BnUC1*, which encodes a nuclear-localized bHLH transcription factor (Figure S1). In addition, it is highly homologous to the *A. thaliana* gene related to curled leaves (*AtCFLAP2*). Cloning and sequencing of *BnUC1* from the mapping parent Zhongshuang 11 (ZS11) and its near-isogenic line ZS11-NIL with up-curled leaves indicated that the gene in ZS11 (named *BnUC1^WT^*) differs from that in ZS11-NIL (named *BnUC1^mut^*) at four nucleotides, but three of these differences do not alter the encoded protein sequence (synonymous mutations). Only the nucleotide substitution at position 599 leads to a change in the encoded protein sequence, with the asparagine (Asn, N) at position 200 substituted with a serine (Ser, S) (N200S) (Figure 1A). An alignment of related sequences from *B. napus*, *Brassica oleracea, Brassica rapa*, and *A. thaliana* (Figure S2) showed that this N200S mutation occurred in a conserved bHLH domain that is required for the formation of homo/heterodimeric bHLH proteins and is probably involved in the binding of bHLH to E-box and G-box promoter elements [30,31]. Thus, this mutation may affect protein functions.

*BnUC1* expression levels in different ZS11 and ZS11-NIL tissues, including the roots, stems, leaves, and flowers, were determined using quantitative real-time polymerase chain reaction (qRT-PCR) technology. *BnUC1* was expressed in various tissues. In ZS11, *BnUC1* was more highly expressed in the roots, leaves, and cotyledons than in the stems and siliques. In ZS11-NIL, the *BnUC1* expression level was higher in the leaves and flowers than in the stems and siliques (Figure 1B). In both materials, *BnUC1* was expressed at relatively high levels in the leaves, indicating that this gene may affect leaf development.

### 2.2. Overexpression of BnUC1^mut^ Decreased the Epidermal Wax Content and Caused Seedling Leaves to Curl Upward

**Figure 2:**
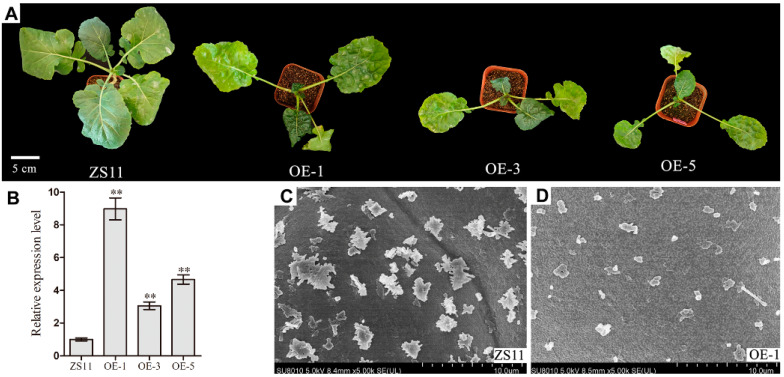
Phenotypes and expression analysis of OE-*BnUC1^mut^* lines. (**A**) Morphology comparison of ZS11 and OE-*BnUC1^mut^* (OE-1, OE-5) lines at seedling stage. Bar = 5 cm. (**B**) Determine the expression level of *BnUC1* gene in OE-*BnUC1^mut^* lines by qRT-PCR. Using *BnActin7* as the internal reference gene. Error bars indicate ± SD (*n* = 3). ** *p* < 0.01. (**C**,**D**): SEM images of cuticle wax crystals on ZS11 (**C**) and OE-1 (**D**) line abaxial sides of leaves in *B. napus*. Bar = 10 μm.

We hypothesized that the mutated *BnUC1* gene expressed at high levels affects leaf morphology and the leaf surface wax content. To test this hypothesis, we constructed a plant transformation vector harboring the full-length *BnUC1^mut^* cDNA sequence under the control of the 35S promoter (35S::*BnUC1^mut^*). The recombinant vector was inserted into the mapping parent ZS11, which has normal flat leaves that are covered with an epidermal cuticle. The genetic transformation completed according to a floral dip method [32] generated seven independent *BnUC1^mut^*-overexpressing lines (OE-*BnUC1^mut^*). The transformed lines were self-pollinated to obtain homozygous T_3_ generation plants (OE-1, OE-3, and OE-5), which had up-curled glossy leaves (Figure 2A). The leaves of the OE-*BnUC1^mut^* lines had decreased surface wax contents. According to a qRT-PCR analysis, *BnUC1^mut^* expression levels were considerably higher in the leaves of the T_3_ generation transgenic lines than in the leaves of ZS11 (Figure 2B). Furthermore, an examination using a scanning electron microscope detected fewer wax crystals on the leaf surface of the transgenic lines than on the leaf surface of ZS11 (Figure 2C,D). Thus, the overexpression of the mutated gene was likely associated with the up-curled glossy leaf phenotype.

**Figure 3:**
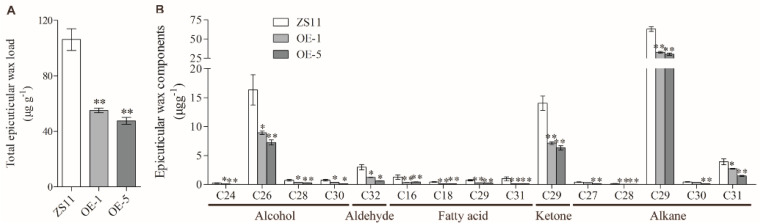
The leaf cuticular wax comparison of ZS11 and OE-*BnUC1^mut^* lines. (**A**) Total wax coverage and amount in leaf epidermis of ZS11 and OE-*BnUC1^mut^* (OE−1 and OE−5) lines. (**B**) Amounts of epidermal wax in leaf epidermis of ZS11 and OE-*BnUC1^mut^* (OE−1 and OE−5) lines. Cuticular wax samples were extracted from seven-week-old plants with chloroform and analyzed using GC-MS. Error bars indicate ± SD from three biological replicates (* *p* < 0.05, ** *p* < 0.01).

The leaf epidermal cuticles of the OE-*BnUC1^mut^* transgenic lines (OE-1 and OE-5) and the wild-type control (ZS11) were extracted and silanized for a quantitative analysis of the leaf wax components using a gas chromatography-mass spectrometry (GC-MS) system. The total epidermal wax contents in leaf on the surface of the OE-1, OE-5, and ZS11 leaves were 55.10, 47.58, and 106.23 μg g^−1^, respectively. Hence, there was a substantial decrease in the total leaf epidermal wax content in the OE lines (Figure 3A). The epidermal cuticle included 15 types of wax components, among which nonacosane (C29 alkane), hexacosanol (C26 alcohol), and 15-nonacosanone (C29 ketone) were the major components (approximately 90% of the total content). The abundances of these major components were consistently much lower at the leaf surface of the transgenic lines overexpressing the mutated gene than at the leaf surface of the control (Figure 3B). Thus, the overexpression of *BnUC1^mut^* appeared to affect the accumulation of cuticle components at the leaf surface, especially the major components.

### 2.3. Knocking out BnUC1^mut^ Restored the Normal Flat Leaf Phenotype

**Figure 4:**
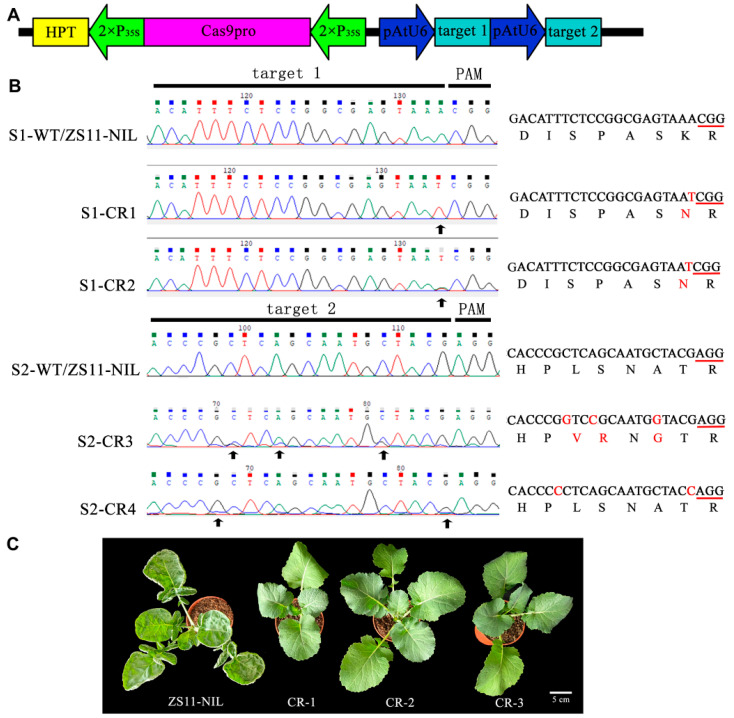
Targets and phenotypes analysis of *BnUC1^mut^* knockout lines. (**A**) The construct of *BnUC1^mut^* CRISPR-Cas9 vector: a hygromycin resistance cassette consisting of the hygromycin phosphotransferase gene (*HPT*) driven by the cauliflower mosaic virus 35S promoter; a Cas9 expression cassette comprising the sequence encoding Cas9 driven by 35S promoter; and two sgRNAs (target1 and target2) driven by the U6 promoters from *Arabidopsis*. (**B**) Four CRISPR-Cas9-induced mutant alleles (named CR-1~4) detected by Sanger sequencing. PAM is indicated by a red underline, while nucleotide mutations are indicated by red letters. (**C**) Morphology of ZS11-NIL and *BnUC1^mut^* knockout lines. Bars = 5 cm.

To further verify the effects of the detected gene mutation, we knocked out *BnUC1^mut^* in ZS11-NIL using a CRISPR/Cas9 approach. A gene-editing vector was designed to target two sites in *BnUC1^mut^* (Figure 4A). A floral dip method was used to transform ZS11-NIL with the gene-editing vector, which generated six transgenic lines. Four homozygous gene-knockout lines (CR-1–4) were obtained following self-pollination (Figure 4B). In both CR-1 and CR-2, the same A-T substitution was detected at target 1, whereas in CR-3 and CR-4, there were three and two base changes at target 2, respectively. The base changes altered the amino acid sequences in CR-1, CR-2, and CR-3, which was in contrast to the synonymous mutation in CR-4. Unlike ZS11-NIL leaves, the leaves of the knockout plants were flat and covered with visible wax (Figure 4C). Therefore, knocking out *BnUC1^mut^* in ZS11-NIL reversed the abnormal leaf morphology of the mutant, resulting in flat leaves that accumulated epidermal wax.

**Figure 5:**
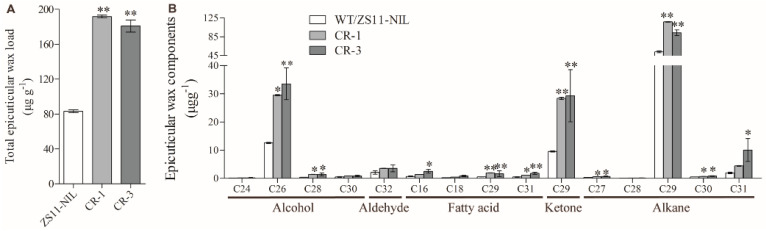
The comparison of leaf cuticular wax components between mapping parent ZS11-NIL and the *BnUC1^mut^* knockout lines. (**A**) Total wax coverage and amount in leaf of ZS11-NIL and *BnUC1^mut^* gene knockout (CR-1 and CR-3) lines. (**B**) Amounts of individual components in leaf of ZS11-NIL and *BnUC1^mut^* gene knockout (CR−1 and CR−3) lines. Cuticular wax samples were extracted from eight-week-old plants with chloroform and analyzed using GC-MS. Error bars indicate ± SD from three biological replicates (* *p* < 0.05, ** *p* < 0.01).

Leaf cuticles were extracted from *BnUC1^mut^* knockout lines (CR-1 and CR-3) and ZS11-NIL and then silanized for a quantitative examination of the leaf epidermal cuticle composition and content. The GC-MS analysis indicated that the total leaf epidermal wax contents in ZS11-NIL, CR-1, and CR-3 were 83.13, 191.62, and 180.83 μg g^−1^, respectively. The total leaf epidermal wax contents in CR-1 and CR-3 were 130.51% and 117.53% higher than the corresponding content in ZS11-NIL, respectively (Figure 5A). Additionally, the contents of the three most abundant components (C29 alkane, C26 alcohol, and C29 ketone) were significantly higher in CR-1 and CR-3 than in ZS11-NIL (Figure 5B). Accordingly, knocking out *BnUC1^mut^* clearly increased the leaf epidermal wax content in *B. napus.* The knockout lines had larger leaf epidermal wax than ZS11-NIL (Figure 5A). Thus, *BnUC1^mut^* overexpression and knockout experiments showed that *BnUC1^mut^* negatively regulates leaf epidermal wax accumulation, leading to the formation of up-curled leaves.

### 2.4. BnUC1^mut^ Downregulates the Expression of VLCFA Biosynthesis-Related Genes

**Figure 6:**
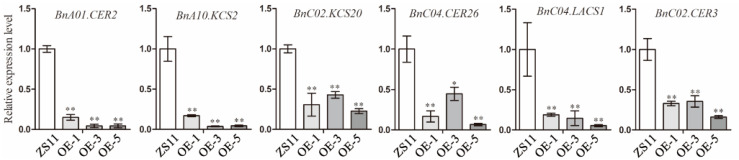
The *BnUC1^mut^* regulates gene expression of long-chain fatty acid biosynthesis. Expression level of six VLCFA biosynthesis genes (*BnA01.CER2/BnaA01G0141100ZS*, BnA10.KCS2/BnaA10G0024400ZS, BnC02.KCS20/BnaC02G0385300ZS, BnC04.CER26/BnaC04G0357800ZS, BnC04.LACS1/BnaC04G0007500ZS, BnC02.CER3/BnaC02G0140500ZS) in *BnUC1^mut^*-overexpressing lines (OE-1, OE-5, and ZS11) and the control ZS11 (* *p* < 0.05, ** *p* < 0.01, *n* = 3).

A transcriptomic analysis of leaves (Table S3) indicated that the expression levels of many genes directly related to VLCFA biosynthesis were significantly downregulated in transgenic lines overexpressing *BnUC1^mut^*. We conducted a qRT-PCR analysis of the transcript levels of these genes. The six selected genes included *ECERIFERUM3* (*CER3*), which encodes an enzyme involved in the production of very-long-chain alkanes (major wax component); the decreased expression of this gene adversely affects cuticular wax biosynthesis [13]. *CER8* encodes long-chain acyl-CoA synthetase 1 (LACS1), which modifies VLCFAs for the synthesis of wax and long-chain (C16) fatty acids [33]. *KCS6* encodes a protein with a major role during the elongation from C26 to C28, making it important for wax synthesis. *KCS5* is a *KCS6* paralog that is also critical for wax biosynthesis [10]. Our qRT-PCR data indicated that the expression levels of these key genes associated with epidermal cuticular wax biosynthesis were significantly downregulated in the leaves of OE-*BnUC1^mut^* lines (Figure 6). Hence, we speculated that the expression of these genes may be regulated by BnUC1.

**Figure 7:**
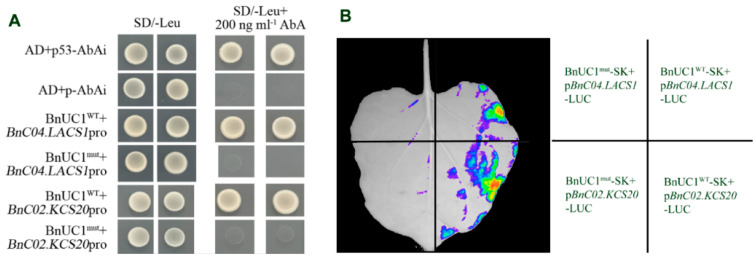
The BnUC1s interact with *LACS1* and *KCS20* gene promoters. (**A**) Observe the growth status of yeast containing two plasmids in SD/-Leu and SD/-Leu+200 ng mL^−1^ AbA (Aureobasidin A) media to determine the interactions between BnUC1^WT^ and BnUC1^mut^ with wax synthesis-related gene promoters, respectively. p53-AbAi+AD and pAbAi+AD were used as positive and negative controls, respectively. (**B**) The luciferase assay showed the binding between BnUC1s and wax synthesis-related gene promoters, respectively, in *N. benthamiana* leaves.

To further investigate whether the changes in the expression of these VLCFA synthesis-related genes are associated with BnUC1^mut^, we performed yeast one-hybrid experiments. Transcription factors belonging to the bHLH family mainly bind to the E-box and palindromic G-box sequences (5′-CANNTG-3′ and 5′-CAGGTG-3′) in the promoters of target genes [30]. Several conserved amino acids in the basic region of the DNA-binding domain of bHLH transcription factors may determine the specificity of the binding to the core consensus sites of different E-box or G-box sequences [34]. We searched the *B. napus* cv ZS11 genome database (http://cbi.hzau.edu.cn/bnapus/synteny/index.php, accessed on 1 September 2024) and detected 37 *LACS* and 76 *KCS* genes. The promoters of 31 *LACS* and 64 *KCS* genes were revealed to contain a G-box motif (Table S1), which serves as a binding site for proteins. Thus, we hypothesized that the bHLH transcription factor BnUC1 influences the expression of major *LACS* and *KCS* genes responsible for wax synthesis, ultimately modulating leaf epidermal cuticle accumulation. To test this hypothesis, the *BnC04.LACS1* (*BnaC04G0007500ZS*) and *BnC02.KCS20* (*BnaC02G0385300ZS*) promoters containing a G-box motif were used to conduct yeast one-hybrid experiments to assess the potential interactions with BnUC1. The yeast one-hybrid results showed that BnUC1^WT^ can bind to the *BnC04.LACS1* and *BnC02.KCS20* promoters, but BnUC1^mut^ cannot (Figure 7A). Consistent with these observations, a luciferase assay showed that BnUC1^WT^, but not BnUC1^mut^, can bind to the *BnC04.LACS1* and *BnC02.KCS20* promoters in *Nicotiana benthamiana* leaves (Figure 7B). Thus, the mutation in *BnUC1^mut^* likely prevents the encoded transcription factor from binding to the promoter of wax synthesis-related genes, leading to a decrease in leaf epidermal wax production and accumulation.

### 2.5. BnUC1 Regulates Lipid Transport to the Leaf Surface

**Figure 8:**
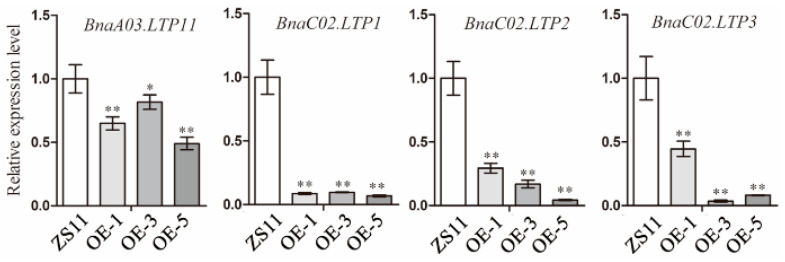
Expression analysis of *LTP* genes in *BnUC1^mut^*-overexpressing and ZS11 lines. Expression level comparison of four *LTP* genes (*BnA03.LTP11*/*BnaA03G0536300ZS*, *BnC02.LTP1*/*BnaC02G0158700ZS*, *BnC02.LTP2*/*BnaC02G0159100ZS*, *BnC02.LTP3*/*BnaC02G054 0400ZS*) in *BnUC1^mut^*-overexpressing lines (OE-1, OE-5, and ZS11) and the control ZS11. Statistical significance of the measurements was determined using Student’s *t* test (* *p* < 0.05, ** *p* < 0.01, *n* = 3).

To further clarify the molecular mechanism underlying the effect of *BnUC1^mut^* on epidermal wax abundance in *B. napus*, leaves from the transgenic lines overexpressing *BnUC1^mut^* and the knockout lines were compared with control leaves at the transcriptome level (Table S3). These comparisons revealed significant changes in the expression levels of multiple *LTP* genes in the overexpression and knockout lines. Some *LTP* genes were expressed at significantly higher levels in the leaves of knockout lines than in ZS11-NIL leaves. In contrast, the expression of some *LTP* genes was downregulated in the OE-*BnUC1^mut^* lines. Therefore, *BnUC1^mut^* may affect the transport of lipids to the leaf surface. To assess this possibility, we first conducted a qRT-PCR analysis of the expression of *LTP* genes in the leaves of OE-*BnUC1^mut^* lines and the control (ZS11). On the basis of the transcriptomic analysis (Table S3), *BnA03.LTP11*, *BnC02.LTP1*, *BnC02.LTP2*, and *BnC02.LTP3* were selected for the qRT-PCR analysis, which indicated that the expression of these genes decreased significantly in the OE-*BnUC1^mut^* leaves (Figure 8). Hence, BnUC1^mut^ may inhibit the accumulation of epidermal wax because of the associated decreased expression of multiple *LTP* genes.

**Figure 9:**
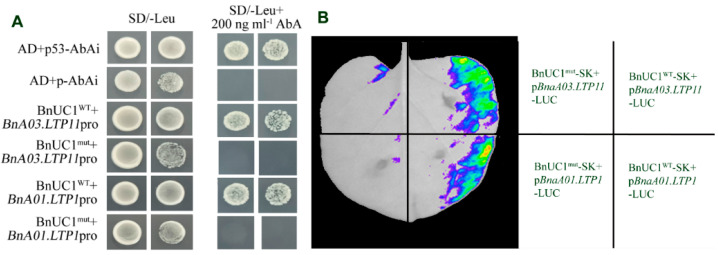
The BnUC1 binding to *LTP* gene promoters. (**A**) Observe the growth status of yeast containing two plasmids in SD/-Leu and SD/-Leu+200 ng/mL AbA (Aureobasidin A) media to determine the interactions between BnUC1^WT^ and BnUC1^mut^ with the *LTP* gene promoters, respectively. The p53−AbAi+AD and pAbAi+AD were used as positive and negative controls, respectively. (**B**) The luciferase assay showed the binding between BnUC1s and the *LTP* gene promoters, respectively, in *N. benthamiana* leaves.

To further elucidate how *LTP* expression levels are regulated by the nuclear-localized transcription factor BnUC1, we performed yeast one-hybrid assays. We analyzed 261 *LTP* promoters in *B. napus* cv ZS11 by applying a bioinformatics approach to screen for E-box or G-box motifs. Of the examined promoters, 189 (i.e., 72.4% of the putative lipid transport-related gene promoters) were revealed to contain the G-box motif (Table S1). Thus, we hypothesized that BnUC1 controls the expression of *LTP* genes responsible for transporting the lipid required for leaf epidermal cuticle accumulation. To assess this hypothesis, we cloned the G-box-containing promoters of two *LTP* genes (*BnaA03.LTP11*/*BnaA03G0536300ZS* and *BnaA01.LTP1/BnaA01G0300300ZS*) that were significantly differentially expressed between ZS11 and OE-*BnUC1^mut^* lines for yeast one-hybrid experiments. Yeast one-hybrid and luciferase assay results showed that BnUC1^WT^ can bind to the *BnaA03.LTP11* and *BnaA01.LTP1* promoters but BnUC1^mut^ cannot (Figure 9A,B). Therefore, the mutated BnUC1 lost its ability to bind to *LTP* promoters, leading to a decrease in the transcription of *LTP* genes in *B. napus* leaves. This is also an important reason for the decreased accumulation of wax on the leaf surface.

### 2.6. BnUC1 Interacts with LTP, MYB, ZFP, and ZIP Proteins

**Figure 10:**
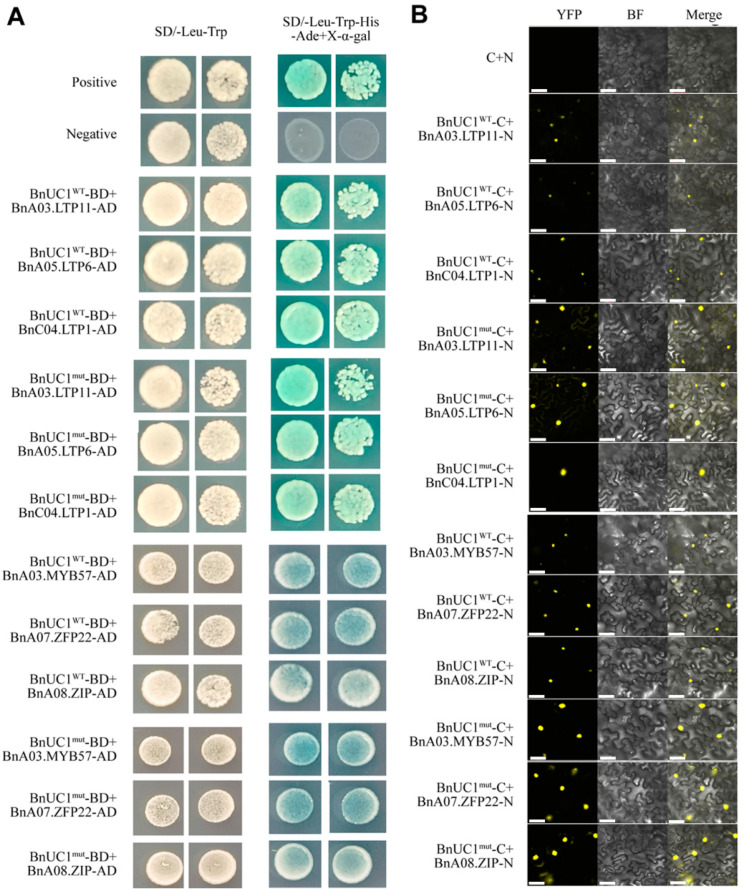
BnUC1^mut^ interacts with BnA03.LTP11, BnA05.LTP6, BnC04.LTP1, BnaA03.MYB57, BnaA07.ZFP22, and BnaA08.ZIP11. (**A**) The point-to-point validation of the protein interaction in yeast cell. The pGADT7-T/pGBKT7-Lam combination was used as the negative control, while the pGADT7-T/pGBKT7-53 combination was used as the positive control. (**B**) The validation of the protein interaction by BiFC assay in tobacco mesophyll cells. The YFP fluorescence and autofluorescence from chloroplasts are indicated in yellow and red, respectively. N, YFP N-terminal; C, YFP C-terminal. The empty vector C+N was used as the negative control. Bars = 20 μm.

Seedlings overexpressing *BnUC1^mut^* had glossy leaves with limited amounts of epidermal wax. Some earlier studies showed that the epidermal wax content is regulated by multiple proteins, including MYB and ZFP transcription factors [19,22,23]. To further explore the mechanism mediating the regulatory effects of BnUC1, yeast two-hybrid experiments were conducted to screen for interactions between BnUC1 and certain proteins, including BnA03.LTP11, BnA05.LTP6, BnC04.LTP1, BnaA03.MYB57, BnaA07.ZFP22, and BnaA08.ZIP11 (Figure 10A). The yeast two-hybrid results indicated that BnUC1 can interact with these proteins, which were selected via a bioinformatics approach. Moreover, there were no obvious differences between BnUC1^WT^ and BnUC1^mut^ in terms of their ability to interact with the selected proteins in yeast. These findings were in accordance with the in vivo protein interactions detected by a bimolecular fluorescence complementation (BiFC) assay (Figure 10B). Hence, these protein interactions play a minor role in epidermal wax accumulation in *B. napus*.

## 3. Discussion

Epidermal wax has crucial functions related to plant responses to various biotic and abiotic factors, such as drought and salinity, pest infestations, light, and pathogen infections [27,33]. Plants contain many bHLH transcription factors with diverse regulatory effects on growth and development [35,36,37]. Although some bHLH transcription factors have been thoroughly investigated [36,38,39], the precise functions of many bHLH transcription factors remain to be determined. In the current study, we identified *BnaA05g18250D* as a gene encoding a bHLH transcription factor that regulates epidermal wax accumulation. We cloned this gene from the mapping parent (ZS11) as well as ZS11-NIL, which has glossy up-curled leaves with relatively little epidermal wax. This gene was renamed *BnUC1*. Notably, this gene is homologous to the *A. thaliana* gene *AtCFLAP2*, which was obtained from a T-DNA insertion mutant and may be related to the mutant phenotype (i.e., up-curled leaves and epidermal wax deficiency); the overexpression of *AtCFLAP2* results in defective leaf cuticles [40]. However, it was unclear how this homolog regulates the accumulation of epidermal wax. Fortunately, we identified a mutated *BnUC1* that encodes a protein with an Asn-to-Ser substitution at conserved domain amino acid position 200. The overexpression of *BnUC1^mut^* resulted in ZS11 seedlings with up-curled leaves and decreased epidermal wax contents (Figure 3). Knocking out *BnUC1^mut^* in ZS11-NIL restored the normal leaf morphology (i.e., flat) (Figure 5). These results suggest that a specific mutation to *BnUC1* is responsible for the glossy and up-curled leaf phenotype in *B. napus*. Our analyses detected clear increases in *BnUC1^mut^* transcription in the leaves of seedlings deficient in epidermal wax in a segregating population, ZS11-NIL [4], and OE-*BnUC1^mut^* lines. These increases in *BnUC1^mut^* expression contributed to the deficiency of epidermal wax in seedling leaves, reflecting the negative regulatory effects of *BnUC1^mut^*.

The single amino acid mutation in BnUC1^mut^ is useful for functionally characterizing BnUC1. Yeast one-hybrid and luciferase assays revealed that BnUC1^WT^ can bind tightly to the promoters of VLCFA biosynthesis-related genes and *LTP* genes, but BnUC1^mut^ cannot (Figure 8 and Figure 10). The mutation of BnUC1 limited the expression level of genes, such as VLCFA biosynthesis genes, which are necessary for producing enough VLCFA for epidermal wax synthesis, and *LTP* genes, which are involved in transporting VLCFA to the leaf surface. A point mutation was detected in a conserved domain associated with bHLH protein dimerization [39]. Our findings imply that this dimerization domain is crucial for the regulation of VLCFA biosynthesis and lipid transport, whereas the basic region at the N-terminus of the bHLH domain is required for the binding to DNA sequences, including E-box (5′-CANNTG-3′) and G-box (5′-CACGTG-3′) motifs in gene promoters [30,41]. In domesticated almond, a mutation in the dimerization domain of bHLH2 prevents the transcription of P450 monooxygenase genes (*PdCYP79D16* and *PdCYP71AN24*) [39]. These observations indicate that this dimerization domain is essential for the binding of bHLH transcription factors to target DNA sequences.

In *A. thaliana*, the bHLH transcription factor AtCFLAP2 can negatively regulate cuticle development via AtCFL1 and the HDZIP IV transcription factor HDG1 [23,35]. According to our findings, BnUC1, which is highly homologous to AtCFLAP2, can interact with LTP, MYB, ZFP, and ZIP proteins, implying that BnUC1 may have multiple positive and negative regulatory effects. This does not contradict the previously reported synergistic mechanism regulating epidermal wax accumulation [35]. The results of our experiments involving BnUC1^mut^ provide direct evidence that BnUC1^WT^ can positively regulate cuticle development.

Curled leaves, which may reflect abnormal leaf development [42], are influenced by various factors, including genes, environmental conditions, and hormones. The development of up-curled leaves may be due to a cuticular wax deficiency in the leaf epidermis, which accelerates transpiration on the adaxial side of leaves and disrupts the balance between transpiration from the adaxial and abaxial sides of leaves. In this study, the formation of up-curled leaves may also be explained by a decrease in the epidermal wax content.

## 4. Materials and Methods

### 4.1. Plant Materials and Growth Conditions

In this study, *B. napus* ZS11 and the near-isogenic line with up-curled leaves (ZS11-NIL) were used as research materials. Both ZS11 and ZS11-NIL were provided by the Rapeseed Genetic Breeding Research Group of Nanjing Agricultural University. The materials required for the experiment are all planted in the experimental field or growth chamber. The temperature in the growth chamber is 22 °C/20 °C, and the illumination time is 16 h/8 h. The seedlings were cultured with vermiculite: nutritious soil (*V*:*V*) = 2:1. After the seedlings grow true leaves, they are transplanted into a square pot (8 cm × 8 cm) or a round pot (diameter of 10 cm). Tobacco plants were grown in a growth chamber at 26 °C (under light) with a 14 h photoperiod. Tobacco plants at the five-leaf stage were used for a subcellular localization analysis and BiFC assays.

### 4.2. Gene Cloning and Sequence Analysis

Total RNA was extracted from ZS11 and ZS11-NIL leaves and then reverse-transcribed to cDNA, which was used as a template for cloning genes. Primers were designed according to the sequences in a rapeseed database (http://www.genoscope.cns.fr/brassicanapus/, accessed on 1 September 2024). Specifically, gene-specific primers for *BnaA05g18250D* (BnUC1-F/R; Table S1) were designed using Primer 5.0. Homologous sequences were retrieved from the NCBI database. Sequences were aligned using ClustalX 2.1 software.

### 4.3. Gene Expression Analysis

The following tissue samples were collected from ZS11 and ZS11-NIL at different developmental stages for an analysis of gene expression patterns: cotyledons, roots, stems, leaves, buds, flowers, and siliques. The collected samples were frozen in liquid nitrogen and stored at −80 °C. Genetically modified seedling leaves were also collected, frozen in liquid nitrogen, and stored at −80 °C. Gene expression was analyzed via qRT-PCR, which was performed using a SYBR Green qPCR SuperMix Kit (TransGen, Beijing, China), with *BnActin7* serving as the internal reference gene. The expression of each gene was examined using three biological replicates. Details regarding the gene-specific qRT-PCR primers are provided in Table S1.

### 4.4. Subcellular Localization

To clarify the subcellular localization of BnUC1, the *BnUC1* coding sequence lacking the terminator was inserted into the p1305-GFP vector to generate the 35S::*BnUC1*-*GFP* construct. The recombinant plasmid and the empty p1305-GFP vector (control) were injected into epidermal cells of tobacco leaves through *Agrobacterium tumefaciens*. The subcellular localization of BnUC1 was determined using a laser confocal microscope.

### 4.5. Expression Vector Construction

The cloned *BnUC1^mut^* sequence was inserted into the overexpression vector pBI121, after which *A. tumefaciens* GV3101 cells were transformed with the recombinant vector. To construct an editing vector, an online program (http://crispr.hzau.edu.cn/CRISPR2/, accessed on 1 September 2024) was used to design two gRNAs targeting *BnUC1^mut^*. The two gRNA sequences were inserted into the pYLCRISPRCas9P35S-H vector. For yeast one-hybrid assays, *BnUC1^WT^* and *BnUC1^mut^* were inserted into separate pGADT7 (AD) vectors. The promoter sequences (1.5 kb) of four genes (*BnC04.LACS1*, *BnC02.KCS20*, *BnaA03.LTP11*, and *BnaA01.LTP1*) were cloned from ZS11 leaves and inserted into the pAbAi vector. p53-AbAi + AD and pAbAi + AD were used as positive and negative controls, respectively. For luciferase assays, the same four gene promoters were inserted into separate pGreen II 0800-LUC vectors, whereas *BnUC1^WT^* and *BnUC1^mut^* were inserted into separate pGreen II 62-SK vectors. To construct BD vectors for yeast hybridization experiments, *BnUC1^WT^* and *BnUC1^mut^* were inserted into separate pGBKT7 vectors. To construct an AD library using SMART technology, RNA was extracted from *B. napus* hypocotyls, leaves, flowers, roots, and pods. To construct BiFC vectors, *BnUC1^WT^* and *BnUC1^mut^* were ligated to C-YFP, whereas *BnA03.LTP11*, *BnA05.LTP6*, *BnC04.LTP1*, *BnaA03.MYB57*, *BnaA07.ZFP22*, and *BnaA08.ZIP11* were ligated to N-YFP.

### 4.6. Wax Extraction and Content Determination

Wax was extracted according to a chloroform immersion method described as follows. Briefly, *B. napus* leaves were soaked thoroughly for 1 min, after which they were transferred to a pre-weighed bottle. The solvent was evaporated using a nitrogen blowing instrument (JHD-001S; Shanghai Jiheng, Shanghai, China), and then the leaf fresh weight and wax weight were recorded. For the wax derivatization reaction, 1 mL of chloroform was added to the dried wax crude extract for re-dissolution, and then 10 μL of N-tetradecane solution (10 μg/μL) was added as an internal standard. The chloroform was dried using nitrogen gas. After adding 40 μL of pyridine and 40 μL of N,O-bis (trimethylsilyl) trifluoroacetamide, the mixture was maintained for 1 h in a water bath set at 70 °C. All reagents were dried using a nitrogen blowing instrument, and then the remaining sample was dissolved in 1.1 mL of chromatography-grade chloroform and stored in a GC sample bottle. A GC-MS analysis involving a DB-5ms capillary column (FS 30 m, 0.25 μM ID, and 0.25 μM df) was performed using the following conditions: EI ion source (70 eV); scanning range, 50–650 m/z; sample inlet temperature, 280 °C; ion source temperature, 250 °C; fourth-stage rod temperature, 150 °C; carrier gas, helium; flow rate, 1.2 mL/min; heating program, increase to 50 °C in 2 min, increase to 200 °C at 40 °C/min, maintain at 200 °C for 2 min, increase to 320 °C at 3 °C/min, and maintain at 320 °C for 30 min.

### 4.7. Data Availability

Primer sequences used in this study are available in Table S1.

### 4.8. Data Analysis

The data were analyzed using SPSS Statistics (version 17) statistical software. Use independent *t*-test to determine the statistical significance of the mean.

## 5. Conclusions

The two *BnUC1* genes (732 bp) cloned from the leaves of ZS11 and ZS11-NIL encode proteins comprising 243 amino acids. The mutation (N200S) in BnUC1^mut^ occurs in the bHLH domain. The overexpression of *BnUC1^mut^* causes leaves to curl upward and have a glossy appearance, which is related to a significant decrease in the epidermal wax content. This overexpression also disrupts the balance in water transpiration from the abaxial and adaxial sides of leaves, ultimately resulting in up-curled leaves. Knocking out *BnUC1^mut^* reversed the up-curled leaf trait, resulting in normal flattened leaves, and significantly increased the epidermal wax content. The N200S mutation in the bHLH domain prevents BnUC1^mut^ from activating the promoters of *LACS1*, *KCS20*, *LTP11*, and *LTP1*. The resulting decrease in the transcription of these genes related to wax synthesis and lipid transport is the main reason for the observed decrease in epidermal wax accumulation and the up-curling of leaves.

## Data Availability

Data are contained within the article and Supplementary Materials.

## Supplementary Materials

The following supporting information can be downloaded at: https://www.mdpi.com/article/10.3390/ijms25179533/s1.

## References

[1] Jetter R., Kunst L., Samuels A.L. (2007). Composition of Plant Cuticular Waxes. Annu. Plant Rev..

[2] Samuels L., Kunst L., Jetter R. (2008). Sealing Plant Surfaces: Cuticular Wax Formation by Epidermal Cells. Annu. Rev. Plant Biol..

[3] Chen G., Komatsuda T., Ma J.F., Li C., Yamaji N., Nevo E. (2011). A Functional Cutin Matrix Is Required for Plant Protection against Water Loss. Plant Signal. Behav..

[4] Yang M., Huang C., Wang M., Fan H., Wan S., Wang Y., He J., Guan R. (2019). Fine Mapping of an Up-Curling Leaf Locus (BnUC1) in Brassica Napus. BMC Plant Biol..

[5] Bernard A., Joubès J. (2013). Arabidopsis Cuticular Waxes: Advances in Synthesis, Export and Regulation. Prog. Lipid Res..

[6] Todd J., Post-Beittenmiller D., Jaworski J.G. (1999). KCS1 Encodes a Fatty Acid Elongase 3-Ketoacyl-CoA Synthase Affecting Wax Biosynthesis in Arabidopsis Thaliana. Plant J..

[7] Fiebig A., Mayfield J.A., Miley N.L., Chau S., Fischer R.L., Preuss D. (2000). Alterations in CER6, a Gene Identical to CUT1, Differentially Affect Long-Chain Lipid Content on the Surface of Pollen and Stems. Plant Cell.

[8] Franke R., Höfer R., Briesen I., Emsermann M., Efremova N., Yephremov A., Schreiber L. (2009). The DAISY Gene from Arabidopsis Encodes a Fatty Acid Elongase Condensing Enzyme Involved in the Biosynthesis of Aliphatic Suberin in Roots and the Chalaza-Micropyle Region of Seeds. Plant J..

[9] Yang K.Z., Jiang M., Wang M., Xue S., Zhu L.L., Wang H.Z., Zou J.J., Lee E.K., Sack F., Le J. (2015). Phosphorylation of Serine 186 of BHLH Transcription Factor SPEECHLESS Promotes Stomatal Development in Arabidopsis. Mol. Plant.

[10] Huang H., Ayaz A., Zheng M., Yang X., Zaman W., Zhao H., Lü S. (2022). Arabidopsis KCS5 and KCS6 Play Redundant Roles in Wax Synthesis. Int. J. Mol. Sci..

[11] Greer S., Wen M., Bird D., Wu X., Samuels L., Kunst L., Jetter R. (2007). The Cytochrome P450 Enzyme CYP96A15 Is the Midchain Alkane Hydroxylase Responsible for Formation of Secondary Alcohols and Ketones in Stem Cuticular Wax of Arabidopsis. Plant Physiol..

[12] Aarts M.G.M., Keijzer C.J., Stiekema W.J., Pereira A. (1995). Molecular Characterization of the CER1 Gene of Arabidopsis Involved in Epicuticular Wax Biosynthesis and Pollen Fertility. Plant Cell.

[13] Kim H., Yu S.I., Jung S.H., Lee B.-h., Suh M.C. (2019). The F-Box Protein SAGL1 and ECeRIFERUM3 Regulate Cuticular Wax Biosynthesis in Response to Changes in Humidity in Arabidopsis. Plant Cell.

[14] Rowland O., Lee R., Franke R., Schreiber L., Kunst L. (2007). The CER3 Wax Biosynthetic Gene from Arabidopsis Thaliana Is Allelic to WAX2/YRE/FLP1. FEBS Lett..

[15] Yang X., Feng T., Li S., Zhao H., Zhao S., Ma C., Jenks M.A., Lü S. (2020). Cer16 Inhibits Post-Transcriptional Gene Silencing of Cer3 to Regulate Alkane Biosynthesis. Plant Physiol..

[16] Xue Y., Xiao S., Kim J., Lung S.C., Chen L., Tanner J.A., Suh M.C., Chye M.L. (2014). Arabidopsis Membrane-Associated Acyl-CoA-Binding Protein ACBP1 Is Involved in Stem Cuticle Formation. J. Exp. Bot..

[17] Pighin J.A., Zheng H., Balakshin L.J., Goodman J.P., Western T.L., Jetter R., Kunst L., Samuels A.L. (2004). Plant Cuticular Lipid Export Requires an ABC Transporter. Science.

[18] DeBono A., Yeats T.H., Rose J.K.C., Bird D., Jetter R., Kunst L., Samuels L. (2009). Arabidopsis LTPG Is a Glycosylphosphatidylinositol-Anchored Lipid Transfer Protein Required for Export of Lipids to the Plant Surface. Plant Cell.

[19] Zhao M., Morohashi K., Hatlestad G., Grotewold E., Lloyd A. (2008). The TTG1-BHLH-MYB Complex Controls Trichome Cell Fate and Patterning through Direct Targeting of Regulatory Loci. Development.

[20] Park C.S., Go Y.S., Suh M.C. (2016). Cuticular Wax Biosynthesis Is Positively Regulated by WRINKLED4, an AP2/ERF-Type Transcription Factor, in Arabidopsis Stems. Plant J..

[21] Kim H., Go Y.S., Suh M.C. (2018). DEWAX2 Transcription Factor Negatively Regulates Cuticular Wax Biosynthesis in Arabidopsis Leaves. Plant Cell Physiol..

[22] Seo P.J., Lee S.B., Suh M.C., Park M.J., Park C.M. (2011). The MYB96 Transcription Factor Regulates Cuticular Wax Biosynthesis under Drought Conditions in Arabidopsis. Plant Cell.

[23] Wu R., Li S., He S., Waßmann F., Yu C., Qin G., Schreiber L., Qu L.J., Gu H. (2011). CFL1, a WW Domain Protein, Regulates Cuticle Development by Modulating the Function of HDG1, a Class IV Homeodomain Transcription Factor, in Rice and Arabidopsis. Plant Cell.

[24] Chalhoub B., Denoeud F., Liu S., Parkin I.A.P., Tang H., Wang X., Chiquet J., Belcram H., Tong C., Samans B. (2014). Early Allopolyploid Evolution in the Post-Neolithic Brassica Napus Oilseed Genome. Science.

[25] Hu J., Chen B., Zhao J., Zhang F., Xie T., Xu K., Gao G., Yan G., Li H., Li L. (2022). Genomic Selection and Genetic Architecture of Agronomic Traits during Modern Rapeseed Breeding. Nat. Genet..

[26] Aharoni A., Dixit S., Jetter R., Thoenes E., Van Arkel G., Pereira A. (2004). The SHINE Clade of AP2 Domain Transcription Factors Activates Wax Biosynthesis, Alters Cuticle Properties, and Confers Drought Tolerance When Overexpressed in Arabidopsis. Plant Cell.

[27] Pu Y., Gao J., Guo Y., Liu T., Zhu L., Xu P., Yi B., Wen J., Tu J., Ma C. (2013). A Novel Dominant Glossy Mutation Causes Suppression of Wax Biosynthesis Pathway and Deficiency of Cuticular Wax in Brassica Napus. BMC Plant Biol..

[28] Liu J., Zhu L., Wang B., Wang H., Khan I., Zhang S., Wen J., Ma C., Dai C., Tu J. (2021). BnA1.CER4 and BnC1.CER4 Are Redundantly Involved in Branched Primary Alcohols in the Cuticle Wax of Brassica Napus. Theor. Appl. Genet..

[29] Wang Y., Jin S., Xu Y., Li S., Zhang S., Yuan Z., Li J., Ni Y. (2020). Overexpression of BnKCS1-1, BnKCS1-2, and BnCER1-2 Promotes Cuticular Wax Production and Increases Drought Tolerance in Brassica Napus. Crop J..

[30] Buck M.J., Atchley W.R. (2003). Phylogenetic Analysis of Plant Basic Helix-Loop-Helix Proteins. J. Mol. Evol..

[31] Liu W., Tai H., Li S., Gao W., Zhao M., Xie C., Li W.X. (2014). BHLH122 Is Important for Drought and Osmotic Stress Resistance in Arabidopsis and in the Repression of ABA Catabolism. New Phytol..

[32] Clough S.J., Bent A.F. (1998). Floral Dip: A Simplified Method for Agrobacterium-Mediated Transformation of Arabidopsis Thaliana. Plant J..

[33] Lü S., Song T., Kosma D.K., Parsons E.P., Rowland O., Jenks M.A. (2009). Arabidopsis CER8 Encodes LONG-CHAIN ACYL-COA SYNTHETASE 1 (LACS1) That Has Overlapping Functions with LACS2 in Plant Wax and Cutin Synthesis. Plant J..

[34] Katayama H., Iwamoto K., Kariya Y., Asakawa T., Kan T., Fukuda H., Ohashi-Ito K. (2015). A Negative Feedback Loop Controlling BHLH Complexes Is Involved in Vascular Cell Division and Differentiation in the Root Apical Meristem. Curr. Biol..

[35] Sharma D., Singh D., Singh K., Dwivedi A., Ranjan A., Sinha A.K. (2023). Phosphorylation of PIF3 by MPK6 Is Required for Coordinated Regulation of MiRNA Biogenesis and Hypocotyl Elongation in Arabidopsis. Environ. Exp. Bot..

[36] Kieffer M., Master V., Waites R., Davies B. (2011). TCP14 and TCP15 Affect Internode Length and Leaf Shape in Arabidopsis. Plant J..

[37] Teng S., Liu Q., Chen G., Chang Y., Cui X., Wu J., Ai P., Sun X., Zhang Z., Lu T. (2023). OsbHLH92, in the Noncanonical Brassinosteroid Signaling Pathway, Positively Regulates Leaf Angle and Grain Weight in Rice. New Phytol..

[38] Song X., Huang Z.N., Duan W.K., Ren J., Liu T.K., Li Y., Hou X.L. (2014). Genome-Wide Analysis of the BHLH Transcription Factor Family in Chinese Cabbage (*Brassica rapa* Ssp. Pekinensis). Mol. Genet. Genom..

[39] Sánchez-Pérez R., Pavan S., Mazzeo R., Moldovan C., Aiese Cigliano R., Del Cueto J., Ricciardi F., Lotti C., Ricciardi L., Dicenta F. (2019). Mutation of a BHLH Transcription Factor Allowed Almond Domestication. Science.

[40] Li S., Wang X., He S., Li J., Huang Q., Imaizumi T., Qu L., Qin G., Qu L.J., Gu H. (2016). CFLAP1 and CFLAP2 Are Two BHLH Transcription Factors Participating in Synergistic Regulation of AtCFL1-Mediated Cuticle Development in Arabidopsis. PLoS Genet..

[41] Murre C., Schonleber P. (1989). A New DNA Binding and Dimerization Motif Inlmmunoglobulin Enhancer MyoD, A&i Myc Proteins. Cell.

[42] Moon J., Hake S. (2011). How a Leaf Gets Its Shape. Curr. Opin. Plant Biol..

