# Supplementary material for: BnUC1 Is a Key Regulator of Epidermal Wax Biosynthesis and Lipid Transport in Brassica napus
Source: Int J Mol Sci. 2024 Sep 2;25(17):9533. doi: 10.3390/ijms25179533 (PMC11394786; doi:10.3390/ijms25179533)
Supplement: Supplementary file 1 [file ijms-25-09533-s001.zip › ijms-3147688-supplementary.pdf]

Supplementary Materials:

Figure S1 Subcellular localization of BnUC1^{WT} and BnUC1^{mut}. Subcellular localization of 35S::BnUC1^{WT}-GFP protein (up), 35S::BnUC1^{mut}-GFP protein (middle) and 35S::GFP protein (down) were expressed in tobacco epidermal cells. Bars=20 μm. 35S::GFP protein (down) showed fluorescence in the nucleus, cytomembrane and cytoplasm. 35S::BnUC1^{WT}-GFP and 35S::BnUC1^{mut}-GFP proteins showed fluorescence in the nucleus. DAPI as a marker for nuclear localization.

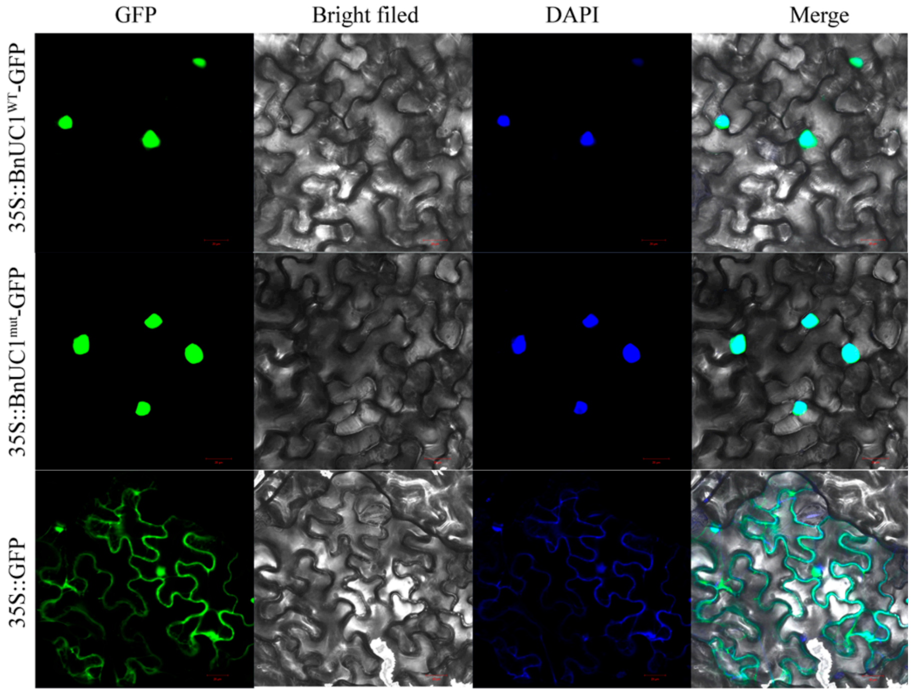

Figure S2 Multiple sequence alignment of BnUC1 and other homologous bHLH protein from *Brassica napus*, *Brassica rapa*, *Brassica oleracea* and *Arabidopsis thealiana*. Black boxes represent conservative bHLH domains.

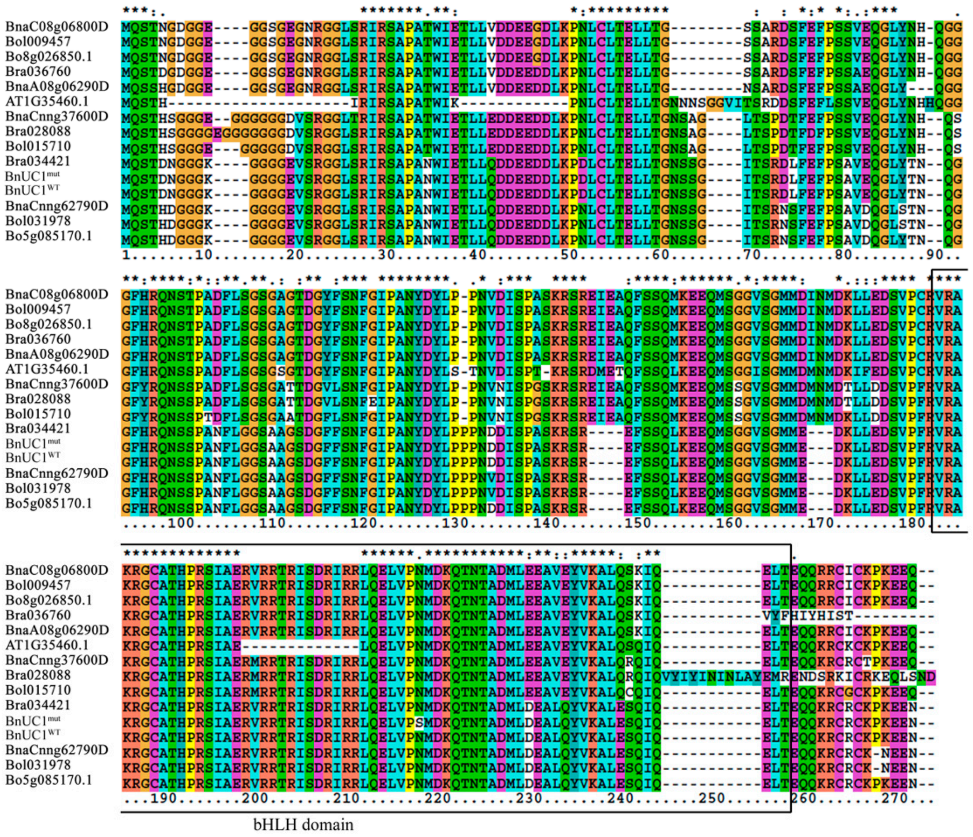

Figure S3 Observation of leaf phenotype between ZS11 and OE-BnUC1^{mut} lines. Morphology comparison of abaxial (A) and adaxial (B) sides leaves between ZS11 and OE-*BnUC1*^{mut} lines at seedling stage. Bar=2 cm.

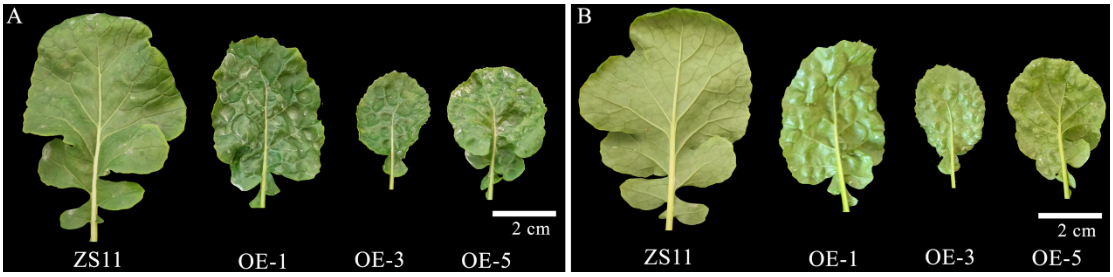

Table S1 List of primers used in this study

Primer names	Primer sequence (5'-3')	Purpose
BnUC1-F	ATGCAGTCCACTGACAAC	Gene clone
BnUC1-R	TTAATTTTCTTCCTTAGGTTTGC	Gene clone
BnUC1-subF	GAACGATAGGGTACCATGCAGTCCACTGACAAC	subcellular localization
BnUC1-subR	GCTCACCATGGATCCATTTTCTTCCTTAGGTTTGC	subcellular localization
BnUC1-VF	CGCGGATCCATGCAGTCCACTGACAAC	Overexpression vector
BnUC1-VR	ACGCGTCGACTTAATTTTCTTCCTTAGGTTTGC	Overexpression vector
35S-F	GAGGCTTACGCAGCAGGTCTCA	Overexpression vector detection
BnUC1-R2	AAGTCACGACTCGTTATTCCG	Overexpression vector detection
BnUC1U-F	CTCCGTTTTACCTGTGGAATCG	Editing vector
BnUC1gR-R	CGGAGGAAAATTCCATCCAC	Editing vector
BnUC1gR-T1	ACATTTCTCCGGCGAGTAAAGTTTTAGAGCTAGAAAT	Editing vector
BnUC1gR-T2	ACCCGCTCAGCAATGCTACGGTTTTAGAGCTAGAAAT	Editing vector
BnUC1U6-T1	TTTACTCGCCGGAGAAATGTCAATCTCTTAGTCGACT	Editing vector
BnUC1U6-T2	CGTAGCATTGCTGAGCGGGTCAATCTCTTAGTCGACT	Editing vector
SP-F	TCGTGCTCCACATGTTGACCG	Editing vector detection
SP-R	ATGCATGCGGCCGCTAGCTC	Editing vector detection
BnUC1-BF	ATGGAGGCCGAATTCATGCAGTCCACTGACAAC	Yeast two hybrid vector
BnUC1-BR	CAGGTCGACGGATCCTTAATTTTCTTCCTTAGGTTTGC	Yeast two hybrid vector
T7	TAATACGACTCACTATAGGGC	Yeast plasmid universal primers
3'AD	AGATGGTGCACGATGCACAG	Yeast plasmid universal primers
3'BD	TAAGAGTCACTTTAAAATTTGTA	Yeast plasmid universal primers
BnUC1-QF	CGCTAACTACGACTACTTGCC	qRT-PCR
BnUC1-QR	CATCCCTGATACACCACCACT	qRT-PCR
BnA01.CER2-QF	AACCCGAACTGAACCCTCAT	qRT-PCR
BnA01.CER2-QR	CATCAACCGGTCGATTTGCT	qRT-PCR
BnaA10.KCS2-QF	AGCGAGGTTGTCTTCTGTGA	qRT-PCR
BnaA10.KCS2-QR	CACCTCCTCCATTTCCACCT	qRT-PCR
BnaC02.CER3-QF	CATTGGAGACGGATGTGCTG	qRT-PCR

BnaC02.CER3-QR	CAAGCTGCCACCATTTCCTT	qRT-PCR
BnaC02.KCS20-QF	ACTTCCTCTTGTCCGTTCGT	qRT-PCR
BnaC02.KCS20-QR	AGGACGGGTGGTGAAGTAAG	qRT-PCR
BnaC04.CER26-QF	GAAGTCCCGCATCGTTACAC	qRT-PCR
BnaC04.CER26-QR	CGCGATATCAAGCAAGCCAT	qRT-PCR
BnaC04.LACS1-QF	GGTGTTGTCTGCCGAAAGTT	qRT-PCR
BnaC04.LACS1-QR	TCGACGTATCCTGCACAAGT	qRT-PCR
BnaA03.LTP11-QF	TGTCACATGAGTACGCCCAT	qRT-PCR
BnaA03.LTP11-QR	TCGATGCGTACGGATGGTTA	qRT-PCR
BnaC02.LTP1-QF	CCAGCGGAAACAAAGGACAA	qRT-PCR
BnaC02.LTP1-QR	GCCACTTCCTTCCCTACCTT	qRT-PCR
BnaC02.LTP2-QF	TGTCCACTCCACCTCAGTTC	qRT-PCR
BnaC02.LTP2-QR	TCTTGAATGTCTCTGCCCGT	qRT-PCR
BnaC02.LTP3-QF	TTCGCAAACAGGTGACCAAG	qRT-PCR
BnaC02.LTP3-QR	TCGAGGAAAGGTTGGGTTGA	qRT-PCR
BnaC02.KCS20-pro-F	TCAAAAGTGCACTATTTTTTTTT	promoter clone
BnaC02.KCS20-pro-R	GGTGAGAACGATGAAAGGGTTAA	promoter clone
BnaC04.LACS1-pro-F	GACTGATAACAAGGGCATCATTA	promoter clone
BnaC04.LACS1-pro-R	TGTAAAAGAAATGGAATAGACAG	promoter clone
BnaA03.LTP11-pro-F	GATCCAGGCTGCAACATAGTGTA	promoter clone
BnaA03.LTP11-pro-R	ATGTATTGTTTACTTGGATGTAC	promoter clone
BnaA01.LTP1-pro-F	TCCGTTGTCAGCCGTAGGAGCAG	promoter clone
BnaA01.LTP1-pro-R	TTCATGTTTCCTCGTGTTTGGAG	promoter clone
BnUC1-AF	GAGGCCAGTGAATTCATGCAGTCCACTGA CAAC	Yeast one hybrid
BnUC1-AR	GAGCTCGATGGATCCTTAATTTTCTTCCTT AGGTTTGC	Yeast one hybrid
BnaC02.KCS20-pro-FAbAi	TTCGAGCTCGGTACCTCAAAAGTGCACTA TTTTTTTTT	Yeast one hybrid
BnaC02.KCS20-pro-RAbAi	TGCCTCGAGGTCGACGGTGAGAACGATGA AAGGGTTAA	Yeast one hybrid
BnaC04.LACS1-pro-FAbAi	TTCGAGCTCGGTACCGACTGATAACAAGG GCATCATTA	Yeast one hybrid
BnaC04.LACS1-pro-RAbAi	TGCCTCGAGGTCGACTGTAAAAGAAATGG AATAGACAG	Yeast one hybrid
BnaA03.LTP11-pro-FAbAi	TTCGAGCTCGGTACCGATCCAGGCTGCAA CATAGTGTA	Yeast one hybrid
BnaA03.LTP11-pro-RAbAi	TGCCTCGAGGTCGACATGTATTGTTTACTT GGATGTAC	Yeast one hybrid
BnaA01.LTP1-pro-FAbAi	TTCGAGCTCGGTACCTCCGTTGTCAGCCGT AGGAGCAG	Yeast one hybrid
BnaA01.LTP1-pro-RAbAi	TGCCTCGAGGTCGACTTCATGTTTCCTCGT GTTTGGAG	Yeast one hybrid

BnUC1-SKF	AGAACTAGTGGATCCATGCAGTCCACTGACAAC	luciferase assay
BnUC1-SKR	CTTGATATCGAATTCTTAATTTTCTTCCTTAGGTTTGC	luciferase assay
BnaC02.KCS20-pro-LUCF	GGCGAATTGGGTACCTCAAAAGTGCACTATTTTTTTTT	luciferase assay
BnaC02.KCS20-pro-LUCR	GTCGACCTCGAGGGGGGTGAGAACGATGAAAGGGTTAA	luciferase assay
BnaC04.LACS1-pro-LUCF	GGCGAATTGGGTACCGACTGATAACAAGGGCATCATTA	luciferase assay
BnaC04.LACS1-pro-LUCR	GTCGACCTCGAGGGGTGTAAAAGAAATGGAATAGACAG	luciferase assay
BnaA03.LTP11-pro-LUCF	GGCGAATTGGGTACCGATCCAGGCTGCAACATAGTGTA	luciferase assay
BnaA03.LTP11-pro-LUCR	GTCGACCTCGAGGGGATGTATTGTTTACTTGGATGTAC	luciferase assay
BnaA01.LTP1-pro-LUCF	GGCGAATTGGGTACCTCCGTTGTCAGCCGTAGGAGCAG	luciferase assay
BnaA01.LTP1-pro-LUCR	GTCGACCTCGAGGGGTTCATGTTTCCTCGTGTTTGGAG	luciferase assay

Table S2 List of *KCS*, *LACS* and *LTP* genes containing G-box elements

The *LACS* homologous genes in *B. napus*.

Gene ID	Gene ID	Gene ID	Gene ID
BnaA01G0134200ZS	*BnaA05G0181300ZS*	*BnaC03G0361100ZS*	*BnaC05G0306100ZS*
BnaA02G0268800ZS	*BnaA05G0470300ZS*	*BnaC03G0461600ZS*	*BnaC05G0531100ZS*
BnaA03G0376800ZS	*BnaA06G0348800ZS*	*BnaC03G0473600ZS*	*BnaC06G0260700ZS*
BnaA03G0382800ZS	*BnaA07G0238900ZS*	*BnaC03G0717000ZS*	*BnaC07G0345700ZS*
BnaA04G0054300ZS	*BnaA09G0139900ZS*	*BnaC04G0007400ZS*	*BnaC07G0478800ZS*
BnaA04G0297500ZS	*BnaC01G0172700ZS*	*BnaC04G0356100ZS*	*BnaC09G0149500ZS*
BnaA05G0006000ZS	*BnaC02G0365600ZS*	*BnaC04G0484200ZS*	*BnaC09G0298300ZS*
BnaA05G0006100ZS	*BnaC03G0266700ZS*	*BnaC04G0614600ZS*	

The *KCS* homologous genes in *B. napus*.

Gene ID	Gene ID	Gene ID	Gene ID
BnaA01G0039300ZS	*BnaA07G0163400ZS*	*BnaC02G0016700ZS*	*BnaC05G0163900ZS*
BnaA02G0016500ZS	*BnaA07G0265100ZS*	*BnaC02G0257000ZS*	*BnaC05G0226200ZS*
BnaA02G0174600ZS	*BnaA07G0275100ZS*	*BnaC02G0490200ZS*	*BnaC06G0296800ZS*
BnaA02G0193200ZS	*BnaA08G0134700ZS*	*BnaC03G0001000ZS*	*BnaC07G0065500ZS*
BnaA02G0365100ZS	*BnaA08G0134800ZS*	*BnaC03G0001100ZS*	*BnaC07G0074300ZS*
BnaA03G0397300ZS	*BnaA08G0316400ZS*	*BnaC03G0493800ZS*	*BnaC07G0237700ZS*
BnaA03G0543500ZS	*BnaA09G0108500ZS*	*BnaC03G0493900ZS*	*BnaC07G0397200ZS*
BnaA04G0173100ZS	*BnaA09G0108600ZS*	*BnaC03G0494000ZS*	*BnaC07G0519900ZS*
BnaA04G0185100ZS	*BnaA09G0572100ZS*	*BnaC03G0745900ZS*	*BnaC07G0520500ZS*
BnaA05G0012000ZS	*BnaA09G0609000ZS*	*BnaC03G0746000ZS*	*BnaC08G0008500ZS*
BnaA06G0045100ZS	*BnaA09G0714600ZS*	*BnaC04G0012500ZS*	*BnaC08G0422900ZS*
BnaA06G0135600ZS	*BnaA10G0007800ZS*	*BnaC04G0195200ZS*	*BnaC08G0462700ZS*
BnaA06G0440600ZS	*BnaA10G0024400ZS*	*BnaC04G0471200ZS*	*BnaC09G0109900ZS*
BnaA07G0037000ZS	*BnaA10G0279300ZS*	*BnaC05G0009400ZS*	*BnaC09G0597100ZS*
BnaA07G0037100ZS	*BnaA10G0282600ZS*	*BnaC05G0025600ZS*	*BnaC09G0600700ZS*
BnaA07G0043200ZS	*BnaC01G0045000ZS*	*BnaC05G0056900ZS*	*Bnascaffold0749G0000100ZS*

The *LTP* homologous genes in *B. napus*.

Gene ID	Gene ID	Gene ID	Gene ID
BnaA01G0065500ZS	*BnaA05G0370500ZS*	*BnaC01G0370600ZS*	*BnaC04G0618000ZS*
BnaA01G0124600ZS	*BnaA05G0458000ZS*	*BnaC01G0406200ZS*	*BnaC04G0618100ZS*
BnaA01G0125400ZS	*BnaA06G0125100ZS*	*BnaC02G0034600ZS*	*BnaC05G0038000ZS*
BnaA01G0201800ZS	*BnaA06G0126300ZS*	*BnaC02G0128700ZS*	*BnaC05G0056300ZS*
BnaA01G0206200ZS	*BnaA06G0292000ZS*	*BnaC02G0140500ZS*	*BnaC05G0153200ZS*
BnaA01G0206300ZS	*BnaA07G0097200ZS*	*BnaC02G0158700ZS*	*BnaC05G0154500ZS*
BnaA01G0250300ZS	*BnaA07G0204900ZS*	*BnaC02G0159200ZS*	*BnaC05G0213200ZS*
BnaA01G0300200ZS	*BnaA07G0255300ZS*	*BnaC02G0206800ZS*	*BnaC05G0239200ZS*
BnaA01G0300300ZS	*BnaA07G0289900ZS*	*BnaC02G0277600ZS*	*BnaC05G0409000ZS*
BnaA01G0328000ZS	*BnaA07G0339200ZS*	*BnaC02G0279000ZS*	*BnaC05G0418200ZS*

BnaA02G0031300ZS	*BnaA07G0339300ZS*	*BnaC02G0279500ZS*	*BnaC06G0086300ZS*
BnaA02G0105900ZS	*BnaA07G0341100ZS*	*BnaC02G0353000ZS*	*BnaC06G0097700ZS*
BnaA02G0115400ZS	*BnaA07G0342200ZS*	*BnaC02G0356100ZS*	*BnaC06G0214400ZS*
BnaA02G0127400ZS	*BnaA08G0007000ZS*	*BnaC02G0372300ZS*	*BnaC06G0398800ZS*
BnaA02G0127600ZS	*BnaA08G0036500ZS*	*BnaC02G0474600ZS*	*BnaC06G0398900ZS*
BnaA02G0161700ZS	*BnaA08G0091200ZS*	*BnaC02G0486900ZS*	*BnaC06G0401300ZS*
BnaA02G0207200ZS	*BnaA08G0126600ZS*	*BnaC02G0540400ZS*	*BnaC06G0402400ZS*
BnaA02G0208500ZS	*BnaA08G0127100ZS*	*BnaC03G0118200ZS*	*BnaC07G0148200ZS*
BnaA02G0208800ZS	*BnaA08G0141200ZS*	*BnaC03G0126500ZS*	*BnaC07G0318400ZS*
BnaA02G0260200ZS	*BnaA08G0142000ZS*	*BnaC03G0140800ZS*	*BnaC07G0393800ZS*
BnaA02G0262600ZS	*BnaA08G0217300ZS*	*BnaC03G0140900ZS*	*BnaC07G0394000ZS*
BnaA02G0273300ZS	*BnaA08G0257800ZS*	*BnaC03G0157800ZS*	*BnaC07G0397000ZS*
BnaA02G0352000ZS	*BnaA09G0116900ZS*	*BnaC03G0211600ZS*	*BnaC07G0441900ZS*
BnaA02G0393700ZS	*BnaA09G0265900ZS*	*BnaC03G0211700ZS*	*BnaC07G0513200ZS*
BnaA02G0407100ZS	*BnaA09G0427500ZS*	*BnaC03G0293700ZS*	*BnaC08G0031500ZS*
BnaA03G0102600ZS	*BnaA09G0481100ZS*	*BnaC03G0370500ZS*	*BnaC08G0044300ZS*
BnaA03G0109700ZS	*BnaA09G0481200ZS*	*BnaC03G0380700ZS*	*BnaC08G0093600ZS*
BnaA03G0109900ZS	*BnaA09G0485700ZS*	*BnaC03G0426100ZS*	*BnaC08G0244600ZS*
BnaA03G0110000ZS	*BnaA09G0614300ZS*	*BnaC03G0428100ZS*	*BnaC08G0317700ZS*
BnaA03G0121400ZS	*BnaA09G0614500ZS*	*BnaC03G0456100ZS*	*BnaC08G0317800ZS*
BnaA03G0121500ZS	*BnaA09G0680500ZS*	*BnaC03G0456200ZS*	*BnaC08G0323300ZS*
BnaA03G0136400ZS	*BnaA09G0721300ZS*	*BnaC03G0485200ZS*	*BnaC08G0470200ZS*
BnaA03G0180700ZS	*BnaA09G0721700ZS*	*BnaC03G0534800ZS*	*BnaC09G0120600ZS*
BnaA03G0308900ZS	*BnaA09G0721800ZS*	*BnaC03G0646900ZS*	*BnaC09G0180800ZS*
BnaA03G0352900ZS	*BnaA10G0037500ZS*	*BnaC03G0754200ZS*	*BnaC09G0309200ZS*
BnaA03G0372900ZS	*BnaA10G0090200ZS*	*BnaC03G0771000ZS*	*BnaC09G0312800ZS*
BnaA03G0373000ZS	*BnaA10G0114400ZS*	*BnaC03G0809300ZS*	*BnaC09G0341900ZS*
BnaA03G0392100ZS	*BnaA10G0124100ZS*	*BnaC04G0048500ZS*	*BnaC09G0380300ZS*
BnaA03G0536300ZS	*BnaA10G0135600ZS*	*BnaC04G0080200ZS*	*BnaC09G0396400ZS*
BnaA04G0242400ZS	*BnaA10G0260400ZS*	*BnaC04G0088000ZS*	*BnaC09G0411500ZS*
BnaA04G0243400ZS	*BnaA10G0271400ZS*	*BnaC04G0089300ZS*	*BnaC09G0427500ZS*
BnaA04G0301200ZS	*BnaC01G0078600ZS*	*BnaC04G0208600ZS*	*BnaC09G0588200ZS*
BnaA04G0301300ZS	*BnaC01G0154900ZS*	*BnaC04G0290800ZS*	*Bnascaffold0026G0005200ZS*
BnaA05G0075700ZS	*BnaC01G0155700ZS*	*BnaC04G0327000ZS*	*Bnascaffold0026G0042000ZS*
BnaA05G0076700ZS	*BnaC01G0259200ZS*	*BnaC04G0329600ZS*	*Bnascaffold0026G0051000ZS*
BnaA05G0183300ZS	*BnaC01G0259300ZS*	*BnaC04G0348100ZS*	
BnaA05G0334500ZS	*BnaC01G0312000ZS*	*BnaC04G0382400ZS*	
BnaA05G0334600ZS	*BnaC01G0370500ZS*	*BnaC04G0558300ZS*	

Table S3 List of DEGs for wax synthesis, transportation, and regulatory factors in transcriptome

The differentially expressed genes between ZS11 and OE-*BnUC1*^{mut} overexpression lines.

Gene ID	logFC	Pvalue	FDR	Gene name	Annotation
**Synthesis**					
BnaC04G0007500ZS	-1.7649	2.9888E-18	3.0761E-16	ACSL1	Long chain acyl-CoA synthetase 1
BnaA05G0006100ZS	2.6489	1.2612E-10	5.9213E-09	ACSL1	Long chain acyl-CoA synthetase 1
BnaC03G0266700ZS	2.8574	0.00045457	0.005734	ACSL1	Long chain acyl-CoA synthetase 1
BnaC02G0140500ZS	-3.3991	2.8169E-39	1.4057E-36	CER3	Very-long-chain aldehyde decarbonylase CER3
BnaC02G0495800ZS	-6.076	1.6442E-19	1.9001E-17	CER26	Protein ECERIFERUM 26-like
BnaA01G0141100ZS	-2.119	1.2274E-09	5.0563E-08	CER2	Protein ECERIFERUM 2
BnaC04G0357800ZS	-1.647	9.5274E-11	4.5775E-09	CER26	Protein ECERIFERUM 26
BnaA02G0369900ZS	1.7474	1.0844E-09	4.5067E-08	CER26	Protein ECERIFERUM 26-like
BnaC03G0193300ZS	2.2154	0.0003624	0.0047308	FAH1	Dihydroceramide fatty acyl 2-hydroxylase FAH1
BnaA10G0023000ZS	1.7061	0.0039667	0.034312	FAO1	Long-chain-alcohol oxidase FAO1
BnaA03G0376200ZS	5.1123	6.694E-13	4.1598E-11	FAO3	Long-chain-alcohol oxidase FAO3
BnaC02G0110800ZS	-8.3284	1.2264E-40	6.8461E-38	FAR4	Probable fatty acyl-CoA reductase 4
BnaA04G0034500ZS	-2.4364	0.00039695	0.005118	FAR6	Fatty acyl-CoA reductase 6
BnaC04G0305900ZS	-2.3103	0.005159	0.041913	FAR6	Fatty acyl-CoA reductase 6
BnaA10G0163900ZS	1.9136	5.5235E-06	0.00012052	FAR1	Fatty acyl-CoA reductase 1
BnaC03G0579500ZS	2.6118	0.0014789	0.015466	FAR4	Probable fatty acyl-CoA reductase 4
BnaA06G0257000ZS	2.9707	0.00024713	0.0034171	FAR4	Probable fatty acyl-CoA reductase 4
BnaA02G0092400ZS	4.2623	4.5862E-11	2.3149E-09	FAR1	Fatty acyl-CoA reductase 1
BnaC06G0323600ZS	-1.7301	0.0035373	0.031322	HHT1	Omega-hydroxypalmitate O-feruloyl transferase
BnaA07G0176000ZS	1.9257	0.0061544	0.048204	HHT1	Omega-hydroxypalmitate O-feruloyl transferase
BnaC06G0169900ZS	2.3721	0.0017399	0.017687	HHT1	Omega-hydroxypalmitate O-feruloyl transferase
BnaC04G0410700ZS	3.6134	8.648E-07	0.000022623	HHT1	Omega-hydroxypalmitate O-feruloyl transferase
BnaA04G0122700ZS	4.4459	2.5032E-09	9.8795E-08	HHT1	Omega-hydroxypalmitate O-feruloyl transferase
BnaA04G0242400ZS	2.9112	0.00037691	0.0048931	CER2	Protein CER1-like 2
BnaC04G0557500ZS	4.22	3.8163E-08	1.2557E-06	CER2	Protein CER1-like 2
BnaC02G0385300ZS	-5.6068	5.7592E-16	4.895E-14	KCS20	3-ketoacyl-CoA synthase 20
BnaC02G0016700ZS	-5.4933	4.1464E-14	2.9496E-12	KCS19	3-ketoacyl-CoA synthase 19
BnaC01G0045000ZS	-2.4689	0.0027413	0.025581	KCS16	3-ketoacyl-CoA synthase 16
BnaA10G0024400ZS	-1.9587	7.6197E-07	0.000020174	KCS2	3-ketoacyl-CoA synthase 2
BnaC09G0109900ZS	2.2159	0.0048999	0.04025	KCS9	3-ketoacyl-CoA synthase 9
BnaA09G0108500ZS	2.4911	0.00005354	0.00091083	KCS9	3-ketoacyl-CoA synthase 9

**Transport**					
BnaC02G0158700ZS	-4.0552	1.7669E-07	5.2473E-06	LTP1	Putative lipid-transfer protein DIR1
BnaC02G0159100ZS	-3.3331	0.000030362	0.00055187	LTP1	Putative lipid-transfer protein DIR1
BnaC02G0345100ZS	-2.8415	0.00050084	0.0062411	LTP1	Putative lipid-binding protein
BnaA09G0628900ZS	-2.224	0.0059358	0.046967	LTP2	Lipid phosphate phosphatase 2
BnaC08G0214600ZS	-1.9686	0.000086114	0.0013866	LTP1	Lipid transfer protein EARLI 1
BnaA08G0280400ZS	-1.9082	0.000012466	0.00025034	LTP1	Lipid transfer protein EARLI 1
BnaA02G0127400ZS	2.0999	0.00062407	0.0074932	LTP1	Putative lipid-transfer protein DIR1
BnaC09G0380300ZS	2.1114	0.0012994	0.01387	LTP1	Putative lipid-transfer protein DIR1
BnaA03G0121400ZS	2.7071	2.1349E-06	0.000050971	LTP1	Putative lipid-transfer protein DIR1
BnaC03G0140800ZS	2.9757	7.9177E-08	2.4859E-06	LTP1	Putative lipid-transfer protein DIR1
BnaA10G0113800ZS	3.4946	9.6148E-10	4.0289E-08	LTP1	Putative lipid-transfer protein DIR1
BnaA02G0127500ZS	3.7383	1.0732E-06	0.000027494	LTP1	Putative lipid-transfer protein DIR1
BnaC02G0540400ZS	-2.6261	0.001383	0.014616	nLTP1	Non-specific lipid-transfer protein-like protein
BnaA03G0317500ZS	-2.4241	2.2976E-06	0.000054441	nLTP6	Non-specific lipid-transfer protein 6
BnaA03G0139800ZS	1.9104	0.000069412	0.0011428	nLTP3	Non-specific lipid-transfer protein 3
BnaA02G0105900ZS	1.9829	0.0010148	0.011301	nLTP4	Non-specific lipid-transfer protein 4
BnaA03G0372900ZS	2.1169	0.0026435	0.024833	nLTP1	Non-specific lipid-transfer protein-like protein
BnaA02G0106000ZS	2.7377	2.2904E-11	1.1945E-09	nLTP3	Non-specific lipid-transfer protein 3
BnaC03G0456100ZS	2.819	0.00016638	0.0024486	nLTP1	Non-specific lipid-transfer protein-like protein
BnaA01G0300300ZS	2.969	0.00015671	0.0023166	nLTP1	Non-specific lipid-transfer protein-like protein
BnaC03G0428100ZS	3.2747	9.6491E-16	7.9949E-14	nLTP2	Non-specific lipid-transfer protein 2
BnaA05G0334600ZS	3.8513	1.0728E-06	0.000027494	nLTP1	Non-specific lipid-transfer protein-like protein
BnaA08G0036500ZS	4.602	2.1692E-58	3.2046E-55	nLTP2	Non-specific lipid-transfer protein 2
BnaA04G0234300ZS	5.0359	2.9463E-19	3.3346E-17	ABCG34	ABC transporter G family member 34
BnaC04G0093200ZS	3.284	0.000039772	0.00070142	ABCG33	ABC transporter G family member 33
BnaA10G0220700ZS	2.9959	6.8482E-06	0.00014603	ABCG6	ABC transporter G family member 6
BnaC04G0372500ZS	2.7759	0.00075396	0.0088135	ABCG19	ABC transporter G family member 19
BnaA06G0104800ZS	2.6267	0.0011535	0.012555	ABCG40	ABC transporter G family member 40
BnaC05G0129800ZS	2.4097	0.00004661	0.00080626	ABCG40	ABC transporter G family member 40
BnaC04G0093300ZS	2.4085	0.0035165	0.031176	ABCG33	ABC transporter G family member 33
BnaA07G0191600ZS	-1.6001	0.00034933	0.0045876	ABCG18	ABC transporter G family member 18
BnaA05G0330500ZS	-1.6239	0.0023863	0.02287	ABCG14	ABC transporter G family member 14
BnaA06G0012300ZS	-2.0038	6.5846E-06	0.00014101	ABCG24	ABC transporter G family member 24
BnaC06G0193700ZS	-2.0124	7.614E-10	3.2483E-08	ABCG18	ABC transporter G family member 18
BnaA09G0510200ZS	-2.2767	5.6241E-08	1.8063E-06	ABCG18	ABC transporter G family member 18
BnaC02G0545200ZS	-2.3642	1.6305E-20	2.0694E-18	ABCF5	ABC transporter F family member 5
BnaC02G0261200ZS	-3.6123	2.6551E-07	7.6473E-06	ABCG25	ABC transporter G family member 25
BnaC02G0424900ZS	-5.8758	7.8943E-37	3.2705E-34	ABCG7	ABC transporter G family member 7

BnaA09G0427600ZS	-1.9611	0.0013531	0.014343	ABCB13	ABC transporter B family member 13
BnaA04G0233700ZS	-3.5077	2.6292E-11	1.3637E-09	ABCE2	ABC transporter E family member 2
BnaC04G0323900ZS	-3.9837	2.1431E-07	6.2659E-06	ABCF4	ABC transporter F family member 4
BnaA09G0194000ZS	-1.7057	0.000045481	0.00078859	ABCI21	ABC transporter I family member 21
**Regulatory factors**					
BnaC01G0485900ZS	-4.4458	3.2705E-09	1.2702E-07	bHLH50	Transcription factor bHLH150
BnaC02G0231400ZS	-3.4287	7.5197E-09	2.7595E-07	bHLH49	Transcription factor bHLH49
BnaC06G0309200ZS	-2.7077	0.00095692	0.010773	bHLH30	Transcription factor bHLH30
BnaA05G0029900ZS	-2.4568	1.8864E-06	0.000045701	bHLH48	Transcription factor bHLH48
BnaC02G0229800ZS	-2.438	0.0031066	0.028335	bHLH30	Transcription factor bHLH30
BnaC01G0240900ZS	-2.4181	0.0033656	0.030146	bHLH147	Transcription factor bHLH147
BnaC09G0002200ZS	-2.3829	0.000042211	0.00073772	bHLH57	Transcription factor bHLH57
BnaA06G0305600ZS	-2.101	6.8677E-08	2.1749E-06	bHLH93	Transcription factor bHLH93
BnaC03G0519300ZS	-1.8925	0.000015303	0.0003001	bHLH93	Transcription factor bHLH93
BnaA09G0019400ZS	-1.8515	0.000036346	0.00064847	bHLH57	Transcription factor bHLH57
BnaC05G0532500ZS	-1.8198	0.000036113	0.00064519	bHLH150	Transcription factor bHLH150
BnaC01G0096900ZS	-1.6582	0.00014266	0.0021357	bHLH68	Transcription factor bHLH68
BnaA01G0064700ZS	1.8977	0.00034934	0.0045876	bHLH69	Transcription factor bHLH69
BnaA03G0367300ZS	2.0073	0.0016823	0.017204	bHLH87	Transcription factor bHLH87
BnaC03G0165400ZS	2.416	0.00005433	0.00092308	bHLH137	Transcription factor bHLH137
BnaA01G0390000ZS	6.2623	2.2421E-20	2.8185E-18	bHLH148	Transcription factor bHLH148
BnaC02G0150100ZS	-3.3517	0.00002528	0.00046842	bHLH041	Putative transcription factor bHLH041
BnaA06G0421100ZS	-2.2965	0.0000172	0.00033254	bHLH28	Transcription factor bHLH28
BnaC09G0243200ZS	2.4867	0.00027981	0.0038065	bHLH28	Transcription factor bHLH28
BnaC01G0233100ZS	-7.3794	2.561E-31	7.5647E-29	HD-ZIP4	Homeobox-leucine zipper protein HAT4
BnaC02G0548400ZS	-6.2038	1.1411E-19	1.3568E-17	HD-ZIP5	Homeobox-leucine zipper protein ATHB-5
BnaC02G0242700ZS	-2.3398	0.004599	0.038541	HD-ZIP13	Homeobox-leucine zipper protein ATHB-13
BnaA04G0149700ZS	-1.706	0.000017223	0.0003328	HD-ZIP9	Homeobox-leucine zipper protein HAT9
BnaA09G0234900ZS	1.6094	0.000027476	0.00050443	HD-ZIP2	Homeobox-leucine zipper protein 2
BnaC04G0611900ZS	1.7944	0.0026015	0.02453	HD-ZIP7	Homeobox-leucine zipper protein ATHB-7
BnaA02G0183900ZS	1.8943	0.00015923	0.0023502	HD-ZIP13	Homeobox-leucine zipper protein ATHB-13
BnaC06G0363500ZS	1.9606	0.00022734	0.003189	HD-ZIP13	Homeobox-leucine zipper protein ATHB-13
BnaA08G0106400ZS	3.9938	4.4474E-09	1.6856E-07	HD-ZIP5	Homeobox-leucine zipper protein HDG4
BnaA09G0213600ZS	4.5283	9.082E-10	3.8251E-08	HD-ZIP2	Homeobox-leucine zipper protein HAT2
BnaA01G0420800ZS	6.0549	6.8235E-19	7.4293E-17	HD-ZIP5	Homeobox-leucine zipper protein HAT5
BnaC02G0319400ZS	-6.0337	6.9185E-19	7.5202E-17	MYB1	Myb family transcription factor APL
BnaC02G0442300ZS	-4.2813	1.5386E-08	5.371E-07	MYB1	Myb family transcription factor EFM
BnaC02G0291200ZS	-3.6592	2.8841E-06	0.000066847	MYB1	L10-interacting MYB domain-containing protein

BnaC02G0239900ZS	-2.2517	0.0020991	0.020576	MYB8	Myb family transcription factor PHL8
BnaC02G0432300ZS	-2.2511	0.0064351	0.049875	MYB1	Putative Myb family transcription factor
Bnascaffold0074G0000200ZS	-2.0503	1.1409E-10	5.3799E-09	MYB13	Myb family transcription factor PHL13
Bnascaffold0163G0000200ZS	-2.0503	1.1409E-10	5.3799E-09	MYB13	Myb family transcription factor PHL13
BnaA02G0320400ZS	1.7409	0.0024415	0.023302	MYB1	Putative Myb family transcription factor
BnaC02G0205500ZS	-7.4541	9.9583E-33	3.2506E-30	MYB75	Transcription factor MYB75
BnaC02G0553100ZS	-6.6438	2.2052E-24	3.7633E-22	MYB44	Transcription factor MYB44
BnaC02G0283700ZS	-5.9477	2.459E-26	4.9083E-24	MYB96	Transcription factor MYB96
BnaC02G0472600ZS	-3.988	2.3367E-07	6.7747E-06	MYB30	Transcription factor MYB30
BnaC02G0255900ZS	-3.8544	2.593E-09	1.0215E-07	MYB6	Transcription repressor MYB6
BnaC02G0154400ZS	-3.5714	5.5297E-06	0.00012061	MYB80	Transcription factor MYB80
BnaC02G0124300ZS	-3.264	0.000046102	0.00079811	MYB59	Transcription factor MYB59
BnaA06G0401300ZS	-3.2612	2.193E-11	1.1474E-09	MYB88	Transcription factor MYB88
BnaC01G0482700ZS	-2.7338	0.0003075	0.0041236	MYB1	Transcription factor MYB1
BnaA06G0357300ZS	-2.7118	0.000001508	0.000037358	MYB111	Transcription factor MYB111
BnaC02G0068100ZS	-2.5418	8.5083E-08	2.6612E-06	MYB16	Transcription factor MYB16
BnaC03G0461400ZS	-2.5399	0.000093896	0.00149	MYB90	Transcription factor MYB90
BnaA03G0227800ZS	-2.522	0.000013051	0.00026107	MYB12	Transcription factor MYB12
BnaC08G0401900ZS	-1.8984	0.0061735	0.048308	MYB17	Transcription factor MYB17
BnaC07G0280300ZS	-1.7089	0.0034859	0.030983	MYB88	Transcription factor MYB88
BnaC01G0497100ZS	-1.7083	1.4566E-06	0.000036178	MYB106	Transcription factor MYB106
BnaA07G0250700ZS	-1.689	0.000046492	0.00080442	MYB96	Transcription factor MYB96
BnaA03G0303900ZS	1.9393	0.0041326	0.035422	MYB108	Transcription factor MYB108
BnaA07G0105500ZS	2.5386	0.00051879	0.0064368	MYB62	Transcription factor MYB62
BnaA03G0283300ZS	2.746	0.000070505	0.001157	MYB57	Transcription factor MYB57
BnaA01G0418000ZS	3.492	9.9141E-06	0.00020363	MYB106	Transcription factor MYB106

The differentially expressed genes between ZS11-NIL and *BnUC1^{mut}* gene knockout lines.

Gene ID	logFC	Pvalue	FDR	Gene name	Annotation
**Synthesis**					
BnaA01G0391800ZS	3.3275	0.0024563	0.0083202	*	Elongation of fatty acids protein 3-like
BnaC03G0193300ZS	3.1794	1.1291E-12	1.8809E-11	FAH1	Dihydroceramide fatty acyl 2-hydroxylase FAH1
BnaC03G0107600ZS	-3.1548	2.1459E-48	1.143E-45	FAR4	Probable fatty acyl-CoA reductase 4
BnaC04G0410700ZS	3.6233	0.00078106	0.0030008	HHT1	Omega-hydroxypalmitate O-feruloyl transferase
BnaA04G0122700ZS	2.9112	0.000085587	0.00040657	HHT1	Omega-hydroxypalmitate O-feruloyl transferase
BnaA09G0108500ZS	-3.2396	0.00027243	0.0011612	KCS	3-ketoacyl-CoA synthase 9
**Transport**					

BnaC02G0158700ZS	3.0515	6.9697E-11	9.0812E-10	LTP1	Putative lipid-transfer protein DIR1
BnaA02G0127500ZS	3.4681	9.354E-10	1.0373E-08	LTP1	Putative lipid-transfer protein DIR1
BnaC02G0159100ZS	3.4721	6.7482E-09	6.586E-08	LTP1	Putative lipid-transfer protein DIR1
BnaC09G0380300ZS	4.1036	2.2138E-11	3.0992E-10	LTP1	Putative lipid-transfer protein DIR1
BnaC09G0379300ZS	4.44	2.6532E-52	1.8793E-49	LTP1	Putative lipid-transfer protein DIR1
BnaA02G0127400ZS	5.0693	1.0861E-11	1.5848E-10	LTP1	Putative lipid-transfer protein DIR1
BnaA10G0114400ZS	5.1543	7.8652E-09	7.5961E-08	LTP1	Putative lipid-transfer protein DIR1
BnaC09G0379200ZS	5.4554	1.3656E-58	1.5951E-55	LTP1	Putative lipid-transfer protein DIR1
BnaA10G0113800ZS	5.4878	8.7214E-48	4.2078E-45	LTP1	Putative lipid-transfer protein DIR1
BnaA03G0352900ZS	3.0501	4.4681E-07	3.2538E-06	nLTP2	Non-specific lipid-transfer protein 2
BnaC05G0418200ZS	3.5879	4.1152E-17	1.1913E-15	nLTP2	Non-specific lipid-transfer protein 2
BnaA08G0036500ZS	5.2596	3.1763E-71	6.6089E-68	nLTP2	Non-specific lipid-transfer protein 2
BnaC03G0428100ZS	5.5322	2.4753E-52	1.7722E-49	nLTP2	Non-specific lipid-transfer protein 2
BnaC03G0162000ZS	2.9563	4.2353E-50	2.611E-47	nLTP3	Non-specific lipid-transfer protein 3
BnaA02G0106000ZS	3.8694	6.4628E-30	7.1479E-28	nLTP3	Non-specific lipid-transfer protein 3
BnaA03G0139800ZS	4.7182	2.5667E-30	2.9313E-28	nLTP3	Non-specific lipid-transfer protein 3
BnaA02G0105900ZS	3.5597	1.6548E-07	1.2951E-06	nLTP4	Non-specific lipid-transfer protein 4
BnaA01G0206200ZS	3.3217	4.8408E-10	5.5917E-09	nLTP5	Non-specific lipid-transfer protein 5
BnaC03G0380700ZS	2.8312	1.848E-10	2.2727E-09	nLTP6	Non-specific lipid-transfer protein 6
BnaA01G0300300ZS	3.0242	0.0067114	0.020151	nLTP1	Non-specific lipid-transfer protein-like protein
BnaA05G0334600ZS	3.2022	0.0017239	0.0060699	nLTP1	Non-specific lipid-transfer protein-like protein
BnaC03G0456100ZS	4.7302	2.5605E-06	0.000016366	nLTP1	Non-specific lipid-transfer protein-like protein
BnaC05G0371400ZS	-3.7937	3.0475E-37	6.8445E-35	ABCC8	ABC transporter C family member 8
BnaA04G0181400ZS	-5.3705	2.4136E-08	2.1528E-07	ABCG3	ABC transporter G family member 3
BnaC04G0496100ZS	-2.8424	0.00033373	0.0013915	ABCG31	ABC transporter G family member 31
BnaC05G0348400ZS	-3.0208	0.000064315	0.00031343	ABCG14	ABC transporter G family member 14
BnaA05G0080200ZS	-3.1747	4.0805E-19	1.5027E-17	ABCG33	ABC transporter G family member 33
BnaC09G0559300ZS	-3.8109	2.0668E-06	0.00001341	ABCF2	ABC transporter F family member 2
BnaA03G0174700ZS	-4.2117	0.000049662	0.00024785	ABCG34	ABC transporter G family member 34
BnaC09G0153200ZS	-3.9205	3.3539E-06	0.000020995	ABCF3	ABC transporter F family member 3
BnaA04G0236900ZS	-4.6378	5.9328E-06	0.000035462	ABCB1	ABC transporter B family member 1
BnaC04G0549600ZS	-3.4836	4.2135E-19	1.5482E-17	ABCE2	ABC transporter E family member 2
BnaC07G0419700ZS	-2.8366	6.6321E-15	1.4724E-13	ABCB9	ABC transporter B family member 9
BnaA03G0203800ZS	-5.3249	6.2814E-08	5.2435E-07	ABCA1	ABC transporter A family member 1
**Regulatory factors**					
BnaC01G0100200ZS	-4.2518	0.000041232	0.0002087	bHLH119	Transcription factor bHLH119

BnaC03G0247000ZS	-2.9113	0.0042225	0.013385	bHLH129	Transcription factor bHLH129
BnaA01G0390000ZS	5.1783	1.9791E-07	1.5288E-06	bHLH148	Transcription factor bHLH148
BnaA01G0395400ZS	4.7692	1.9401E-06	0.000012653	bHLH150	Transcription factor bHLH150
BnaC07G0434900ZS	4.1682	0.000073152	0.00035237	bHLH162	Transcription factor bHLH162
BnaC03G0486200ZS	4.2049	0.000057352	0.00028225	bHLH25	Transcription factor bHLH25
BnaA09G0210100ZS	-3.3931	0.001932	0.0067205	bHLH28	Transcription factor bHLH28
BnaA05G0029900ZS	-2.9052	7.712E-09	7.4612E-08	bHLH48	Transcription factor bHLH48
BnaC05G0517800ZS	-3.0462	6.4077E-08	5.3416E-07	bHLH62	Transcription factor bHLH62
BnaC01G0042600ZS	-3.9334	0.00023262	0.0010076	bHLH63	Transcription factor bHLH63
BnaA01G0037200ZS	-3.5527	0.000037805	0.00019288	bHLH63	Transcription factor bHLH63
BnaA06G0422700ZS	-3.3117	5.5882E-07	3.9939E-06	bHLH71	Transcription factor bHLH71
BnaA09G0659200ZS	-3.1029	1.567E-07	0.000001231	bHLH74	Transcription factor bHLH74
BnaA03G0367300ZS	-3.232	1.7141E-17	5.1994E-16	bHLH87	Transcription factor bHLH87
BnaC03G0448300ZS	-3.1314	5.5922E-12	8.4544E-11	bHLH87	Transcription factor bHLH87
BnaC08G0518400ZS	-5.3307	3.5056E-08	3.0455E-07	bHLH90	Transcription factor bHLH90
BnaA09G0005500ZS	7.6912	1.0321E-19	4.0902E-18	HD-ZIP2	Homeobox-leucine zipper protein ANTHOCYANINLESS 2
BnaA05G0012400ZS	-3.2266	0.0034081	0.011109	HD-ZIP7	Homeobox-leucine zipper protein ATHB-7
BnaA09G0213600ZS	3.942	8.7799E-06	0.000050961	HD-ZIP2	Homeobox-leucine zipper protein HAT2
BnaA01G0420800ZS	5.9234	2.0943E-10	2.5605E-09	HD-ZIP5	Homeobox-leucine zipper protein HAT5
BnaC06G0397700ZS	-3.3651	1.5843E-09	1.701E-08	HD-ZIP11	Homeobox-leucine zipper protein HDG11
BnaC02G0265200ZS	3.2255	0.003421	0.011142	HD-ZIP11	Homeobox-leucine zipper protein HDG11
BnaC08G0154500ZS	-2.8684	0.000001188	8.0381E-06	HD-ZIP4	Homeobox-leucine zipper protein HDG4
BnaA01G0150700ZS	-5.3742	2.2683E-08	2.0335E-07	HD-ZIP6	Homeobox-leucine zipper protein HDG6
BnaC02G0319400ZS	-3.5609	1.4901E-16	4.0545E-15	MYB1	Myb family transcription factor APL
BnaA02G0327900ZS	-3.1972	1.5167E-08	1.3934E-07	MYB1	Myb family transcription factor EFM
BnaC07G0284400ZS	-3.1763	2.9632E-07	2.2251E-06	MYB1	Myb family transcription factor EFM
Bnascaffold0163G0000200ZS	-3.3857	5.717E-21	2.5876E-19	MYB13	Myb family transcription factor PHL13
Bnascaffold0074G0000200ZS	-3.3857	5.717E-21	2.5876E-19	MYB13	Myb family transcription factor PHL13
Bnascaffold0109G0000100ZS	-3.0786	0.0055883	0.017158	MYB13	Myb family transcription factor PHL13
BnaA02G0023600ZS	-2.8319	0.012039	0.03351	MYB5	Myb family transcription factor PHL5
BnaA03G0146900ZS	2.9878	0.0073733	0.021892	MYB2	MYB-like transcription factor ETC2
BnaC01G0482700ZS	-3.5834	0.000045069	0.00022661	MYB1	Transcription factor MYB1

BnaA09G0612500ZS	4.6105	6.1987E-06	0.000036929	MYB106	Transcription factor MYB106
BnaA01G0418000ZS	5.2808	5.271E-08	4.4536E-07	MYB106	Transcription factor MYB106
BnaA06G0093300ZS	-4.2694	0.000045888	0.00023043	MYB124	Transcription factor MYB124
BnaA07G0079700ZS	3.1872	1.0427E-06	7.1254E-06	MYB15	Transcription factor MYB15
BnaC07G0381200ZS	-2.841	4.5137E-06	0.000027558	MYB28	Transcription factor MYB28
BnaC07G0316500ZS	-3.1564	0.0044492	0.014012	MYB30	Transcription factor MYB30
BnaA05G0442000ZS	-2.8391	0.0053789	0.016585	MYB3	Transcription factor MYB3R-3
BnaA06G0183200ZS	-2.97	1.1401E-10	1.4418E-09	MYB48	Transcription factor MYB48
BnaC03G0607300ZS	-2.8835	1.1677E-15	2.8521E-14	MYB48	Transcription factor MYB48
BnaC09G0432300ZS	-2.9408	2.2212E-09	2.3297E-08	MYB59	Transcription factor MYB59
BnaC04G0007900ZS	4.2614	1.2583E-09	1.3719E-08	MYB78	Transcription factor MYB78
BnaA08G0255600ZS	-3.4356	1.8589E-10	2.2853E-09	MYB80	Transcription factor MYB80
BnaC05G0157200ZS	2.8484	0.011196	0.031452	MYB86	Transcription factor MYB86
BnaC08G0467300ZS	3.0113	0.00041609	0.0016978	MYB86	Transcription factor MYB86
BnaA02G0325000ZS	-3.0746	0.00072327	0.0027996	MYB88	Transcription factor MYB88
BnaA07G0287000ZS	5.7831	2.0731E-12	3.3235E-11	MYB90	Transcription factor MYB90
BnaA07G0250700ZS	-3.3212	3.7633E-15	8.5779E-14	MYB96	Transcription factor MYB96
BnaC06G0276600ZS	-2.9381	1.0165E-12	1.7056E-11	MYB96	Transcription factor MYB96
BnaA02G0400500ZS	-2.9194	4.3363E-10	5.0457E-09	MYB96	Transcription factor MYB96
Bnascaffold1095G0000200ZS	-2.9194	4.3363E-10	5.0457E-09	MYB96	Transcription factor MYB96
